# Mitochondrial-based therapies for neurodegenerative diseases: a review of the current literature

**DOI:** 10.1007/s00210-025-04014-0

**Published:** 2025-03-31

**Authors:** Al-Hassan Soliman Wadan, Ahmed H. Shaaban, Mohamed Z. El-Sadek, Salah Abdelfatah Mostafa, Ahmed Sherief Moshref, Ahmed El-Hussein, Doha El-Sayed Ellakwa, Samah S. Mehanny

**Affiliations:** 1 Department of Oral Biology, Faculty of Dentistry, Galala University, Galala Plateau, Attaka, Suez Governorate 15888 Egypt; 2 Department of Biology, Faculty of Science, Galala University, Galala Plateau, Attaka,, Suez Governorate 15888 Egypt; 3 Biotechnology Program, Faculty of Agriculture, Ain Shams University, Ain Shams, Egypt; 4 Faculty of Dentistry, Galala University, Galala Plateau, Attaka, Suez Governorate 15888 Egypt; 5 Department of Laser Applications in Meteorology, Photochemistry, and Biotechnology, The National Institute of Laser Enhanced Science, Cairo University, Cairo, 11316 Egypt; 6 Department of Biochemistry and Molecular Biology, Faculty of Pharmacy for Girls, Al-Azhar University, Cairo, Egypt; 7 Department of Biochemistry, Faculty of Pharmacy, Sinai University, Kantra Branch, Ismailia, Egypt; 8 Department of Oral Biology, Faculty of Dentistry, Cairo University, Cairo, Egypt

**Keywords:** Neurodegenerative diseases, Therapeutic strategies, Mitochondrial dysfunction, Oxidative stress, Mitochondria-focused therapies

## Abstract

Neurodegenerative disorders present significant challenges to modern medicine because of their complex etiology, pathogenesis, and progressive nature, which complicate practical treatment approaches. Mitochondrial dysfunction is an important contributor to the pathophysiology of various neurodegenerative illnesses, including Alzheimer’s disease (AD), Parkinson’s disease (PD), and amyotrophic lateral sclerosis (ALS). This review paper examines the current literature highlighting the multifaceted functions of mitochondria, including energy production, calcium signaling, apoptosis regulation, mitochondrial biogenesis, mitochondrial dynamics, axonal transport, endoplasmic reticulum–mitochondrial interactions, mitophagy, mitochondrial proteostasis, and their crucial involvement in neuronal health. The literature emphasizes the increasing recognition of mitochondrial dysfunction as a critical factor in the progression of neurodegenerative disorders, marking a shift from traditional symptom management to innovative mitochondrial-based therapies. By discussing mitochondrial mechanisms, including mitochondrial quality control (MQC) processes and the impact of oxidative stress, this review highlights the need for novel therapeutic strategies to restore mitochondrial function, protect neuronal connections and integrity, and slow disease progression. This comprehensive review aims to provide insights into potential interventions that could transform the treatment landscape for neurodegenerative diseases, addressing symptoms and underlying pathophysiological changes.

## Introduction

Millions of individuals worldwide suffer from neurodegenerative disorders like Alzheimer’s disease (AD), Parkinson’s disease (PD), and amyotrophic lateral sclerosis (ALS), which progressively impair memory, mobility, independence, and quality of life (Wadan et al. 2024). As our global population ages, these illnesses become increasingly widespread, imposing a significant emotional and financial strain on individuals, caregivers, and healthcare systems (Feigin et al. 2019; Scheltens et al. 2021; Breijyeh and Karaman 2020). For decades, the quest for treatments has primarily focused on symptom management rather than preventing or curing underlying damage. This reality highlights the critical need for novel therapies that address the underlying causes of these catastrophic conditions rather than simply masking their consequences (Zamanian et al. 2016; Zamanian et al. 2023).

**Fig. 1:**
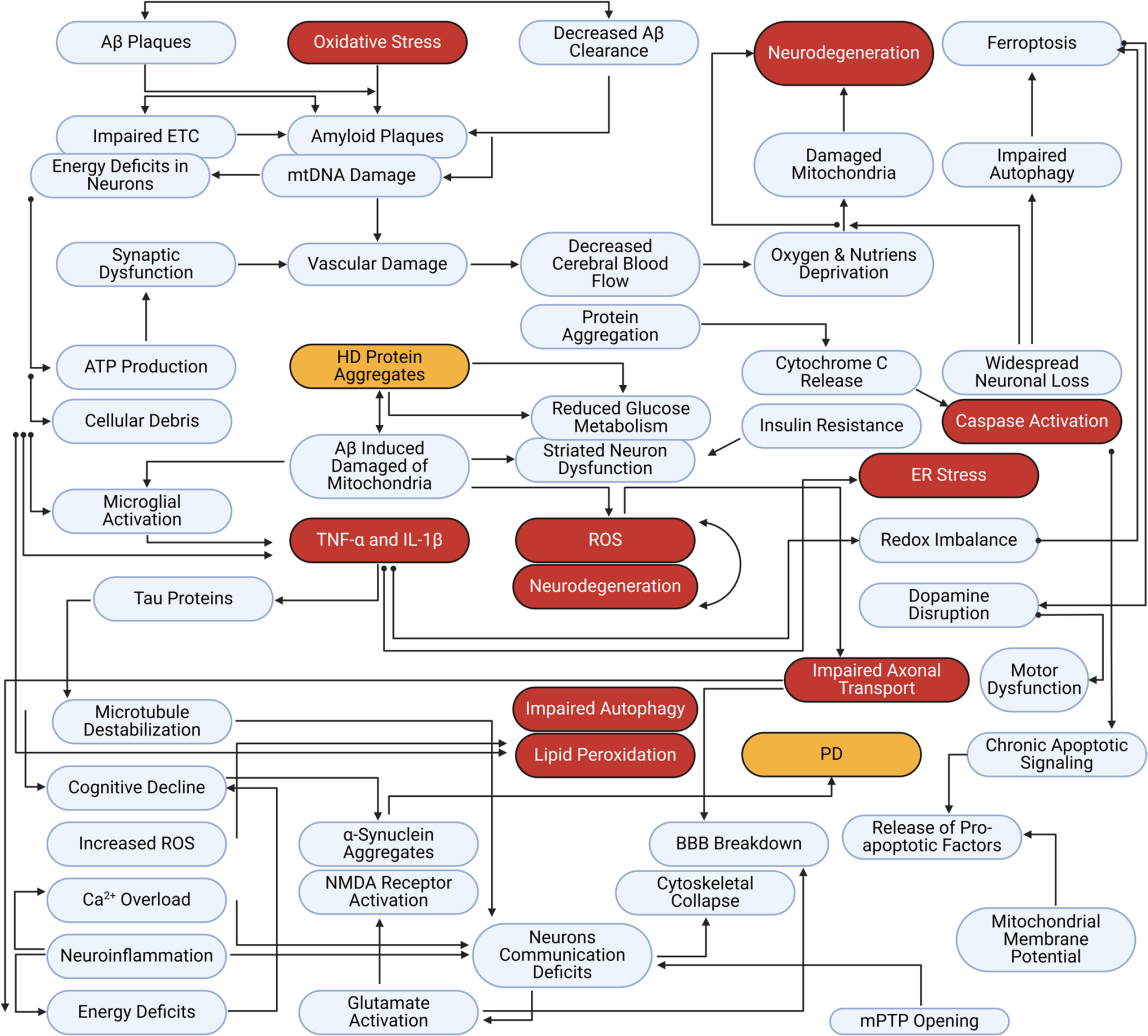
An overall illustration of all cellular and molecular pathways involved in neurodegenerative and inflammation cascades

The role of mitochondria, the microscopic powerhouses within our cells, is a hot topic of discussion. Mitochondria are more than just energy providers; they are vital for energy production, calcium signaling, and regulating programmed cell death pathways (Fig. 1) (Nunnari and Suomalainen 2012; Lin and Beal 2006). Due to their elevated energy requirements, neurons are vulnerable to mitochondrial impairment. When mitochondria fail, brain cells begin to suffer, laying the groundwork for the progressive loss of function seen in many neurodegenerative illnesses. Thus, it is becoming evident that protecting and restoring mitochondrial health may be critical to maintaining neuron integrity and halting the inexorable course of these diseases.

**Fig. 2:**
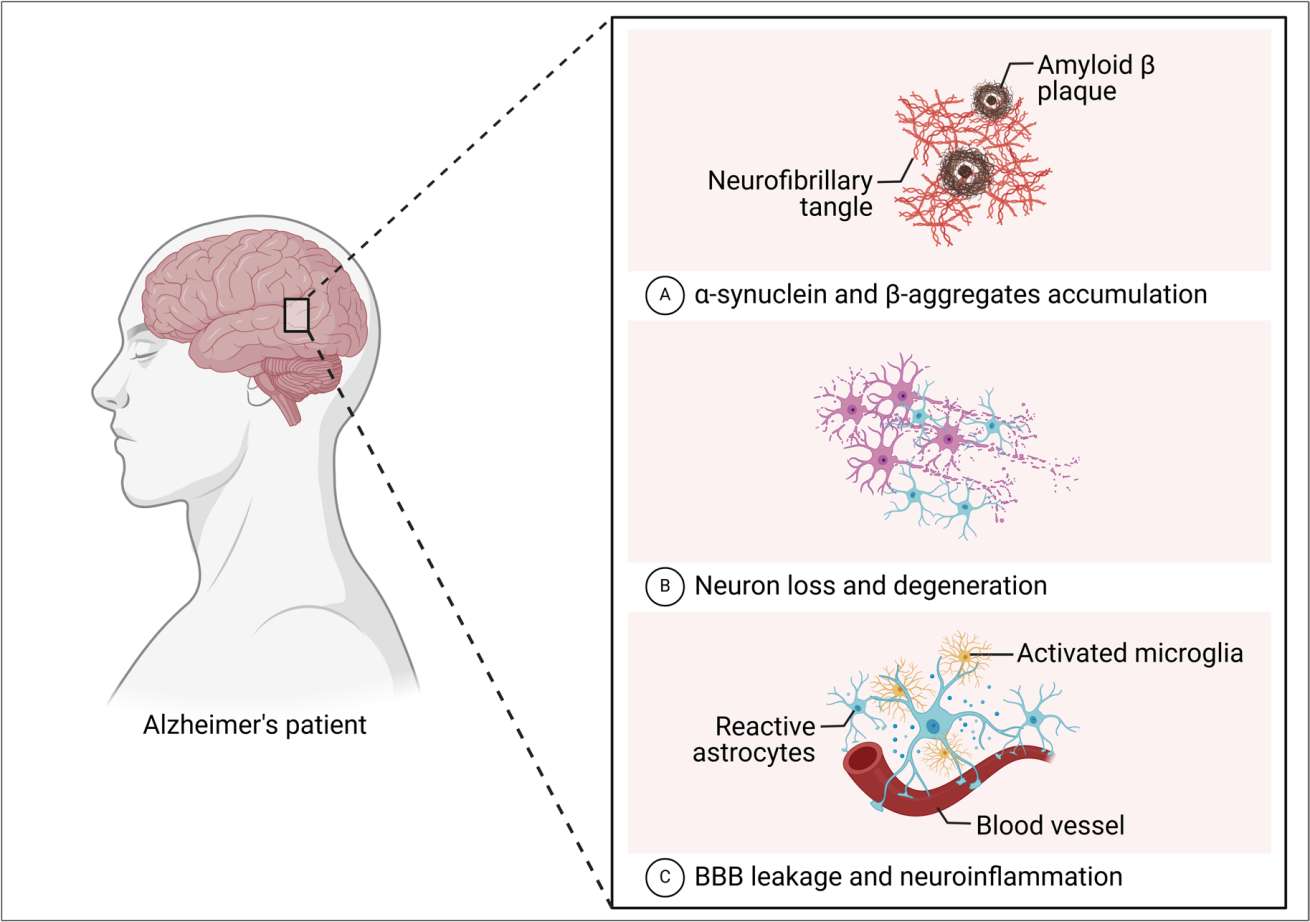
Neuropathological features of neurodegenerative diseases. **A** The neurons are characterized by α-synuclein accumulation and β-amyloid aggregation. **B** Additionally, they are characterized by neuron loss and myelin degeneration. **C** The blood-brain barriers are inflamed and leaking, and there is localized neuroinflammation

In neurodegenerative diseases, where hallmark features include the buildup of amyloid-β plaques and tau protein tangles, mitochondria are typically neglected but play a critical role (Fig. 2) (Swerdlow 2018; Reddy and Oliver 2019; Wang et al. 2008). Dysfunctional mitochondria generate less energy and more toxic ROS, which can injure critical molecules and impair synaptic connections, gradually weakening cognitive functioning, such as memory and learning. In this review, we analyze the current research trends in targeted therapies to enhance bioavailability and efficacy while mitigating potential adverse effects of the general application of antioxidants (Meulmeester et al. 2022). Moreover, this review aims to provide insights into future directions for developing effective mitochondrial-based interventions in neurodegenerative diseases by understanding these therapeutic strategies.

## Neurodegenerative diseases

**Fig. 3:**
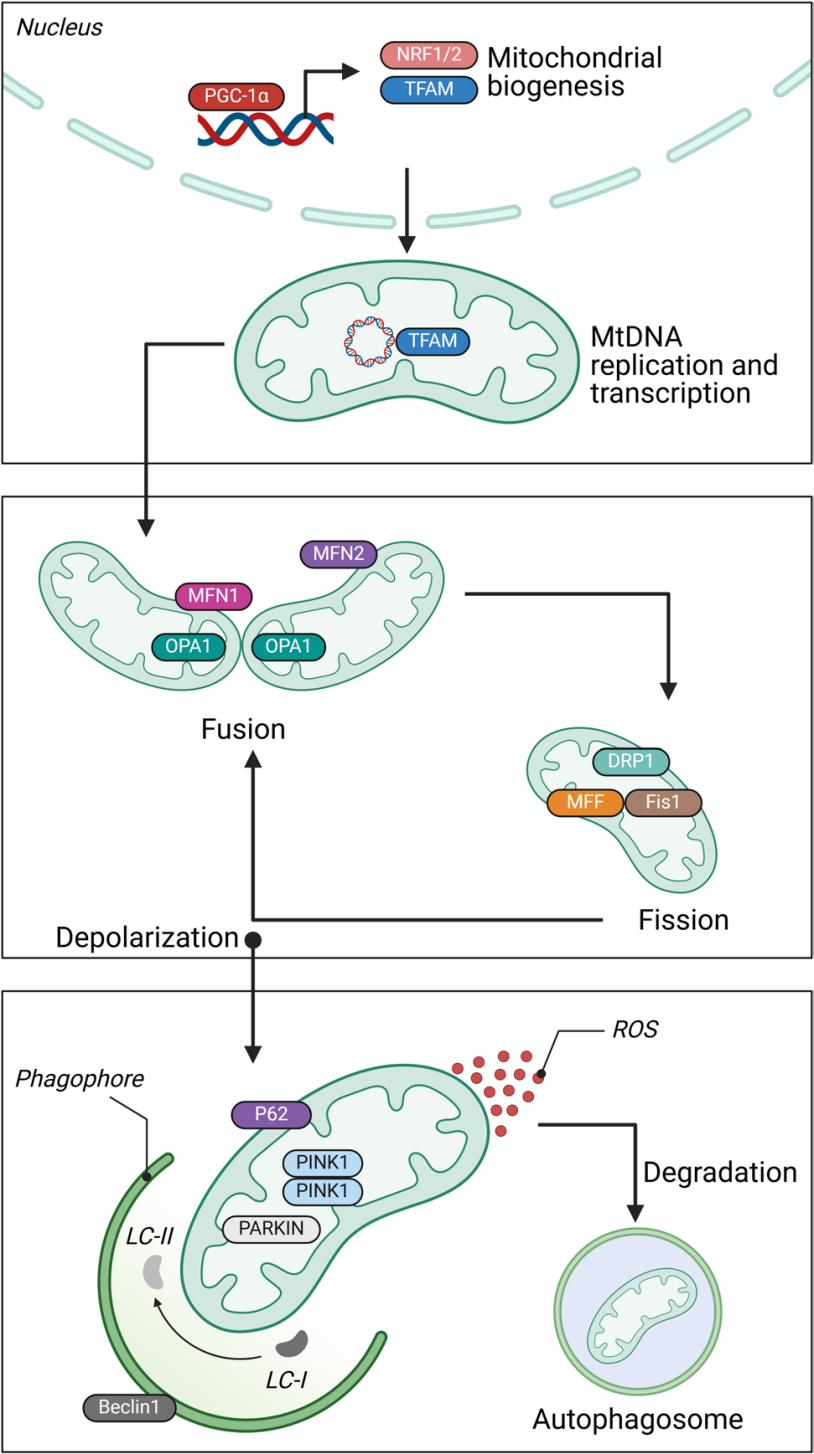
The mechanisms of MQC encompass multiple interconnected processes. At the core of mitochondrial biogenesis is PGC-1α, which functions as the central regulator by activating the expression of nuclear genes, including NRF1, NRF2, and TFAM. Mitochondria undergo a dynamic life cycle involving fusion and fission. Fusion facilitates the creation of interconnected, extended mitochondrial networks, while fission results in smaller, fragmented mitochondria. The proteins mitofusins (MFN1 and MFN2) and optic atrophy 1 (OPA1) promote mitochondrial membrane fusion, whereas DRP1, FIS1, and MFF induce mitochondrial fission. Over time, mitochondria experience oxidative damage, particularly under conditions such as CVD. Fission is essential for the segregation and elimination of impaired mitochondria. This clearance process is mitophagy, which begins with accumulating PINK1 kinase on the outer mitochondrial membrane. PINK1 then recruits PARKIN, a protein that marks the damaged mitochondria for degradation via the autophagosome. The protein p62 facilitates the transport of mitochondria to the autophagosome, which is subsequently degraded during active autophagy. The autophagosome’s development depends on Beclin1 and the transformation of LC3-I to LC3-II via its association with phosphatidylethanolamine, a key step in autophagic vesicle maturation

PD is most known for its tremors, rigidity, and difficulties moving, but the mitochondrial breakdown is also a significant cause. The dopaminergic neurons in the substantia nigra—the brain region most afflicted by Parkinson’s disease—are highly susceptible to mitochondrial stress (Exner et al. 2012; Pickrell and Youle 2015; Ryan et al. 2015). Mutations in genes like PINK1 and PARKIN disrupt mitochondrial quality control (MQC) (Fig. 3). This gap causes an accumulation of defective mitochondria, resulting in a deadly loop of increased ROS and decreased energy production, eventually threatening the neurons that govern movement.

A similar pattern develops with ALS, a disease affecting the motor neurons that connect the brain and spinal cord to muscles. In ALS, genetic and environmental variables combine to impair mitochondrial function, triggering a cascade of cellular distress (Manfredi and Xu 2005; Vande Velde et al. 2011; Cozzolino et al. 2013). Damaged mitochondria fail to meet the continual energy requirements of these long, sophisticated nerve cells. As neurons deteriorate, they cannot effectively transfer signals to muscles, resulting in gradual weakening and eventual paralysis.

Given these discoveries, scientists are now considering “mitochondrial-based therapies” as a revolutionary new strategy for treating neurodegenerative diseases (Alshial et al. 2023; Mohamed et al. 2023; Wadan et al. 2024). These approaches target underlying mitochondrial metabolic and oxidative dysfunction rather than focusing solely on downstream damage (Onyango et al. 2016; Swerdlow 2012; Johri and Beal 2012a, b). Improving mitochondrial function may slow disease progression and restore or maintain neuronal health in ways traditional medications cannot. For instance, one interesting strategy is to use antioxidants that target the mitochondria. Unlike ordinary antioxidants, which circulate throughout the cell, these specialized chemicals, such as MitoQ and SkQ1, target the source of the problem—the mitochondrial matrix (Smith and Murphy 2010; Skulachev et al. 2017). These tailored antioxidants can prevent neuronal harm by neutralizing ROS at their source. Early research indicates that these treatments may help neurons maintain their activities longer by preventing mitochondria from ROS-induced damage.

Other efforts aim to enhance the synthesis of new, healthier mitochondria. Molecules that activate transcriptional coactivators, such as PGC-1α, can promote mitochondrial biogenesis (Wenz 2013; Austin and St-Pierre 2012). This method may enable neurons to maintain higher levels of function and resilience than continuous illness processes. Finally, the goal is that by improving the quantity and quality of mitochondria, neurons’ metabolic load caused by neurodegenerative disorders could be triggered and enhanced. A related technique involves repairing the cell’s mitochondrial recycling mechanism, known as mitophagy. Typically, cells use mitophagy to eliminate old, damaged mitochondria (Youle and Van der Bliek 2012; Pickles et al. 2018). However, this system fails in neurodegenerative diseases. Researchers assist cells in cleaning out malfunctioning mitochondria by designing medications that restore normal mitophagy, increasing overall mitochondrial network health and neuron survival (Chaudhary et al. 2023).

Beyond medications, novel and innovative treatments are emerging. Mitochondrial transplantation, for example, entails putting healthy mitochondria into damaged cells to restore energy metabolism. Although still in its early stages, preliminary investigations in animal models suggest that this technique could help mend cells harmed by faulty mitochondria (Masoudi et al. 2020; Chang et al. 2019). Another frontier is gene therapy: Utilizing precise gene-editing methods such as CRISPR/Cas9, scientists may soon be able to fix mitochondrial DNA abnormalities that cause specific neurodegenerative diseases (Gammage and Viscomi 2021; Moraes 2014). While these concepts are fantastic, making them a reality is no minor feat. The human brain is highly complex, and neurodegenerative illnesses are frequently caused by a network of interrelated pathways that extend beyond mitochondrial malfunction. Delivering medications properly to neurons, guaranteeing therapeutic safety, and demonstrating long-term efficacy are all substantial challenges (Onyango and Khan 2018; Costa and Scorrano 2012). However, it is precisely this intricacy that highlights the significance of these methods. Instead of simply concealing symptoms, mitochondrial-based medicines address the underlying reasons for neuronal deterioration, providing authentic optimism that one day, we will be able not only to halt but possibly reverse the progression of these awful diseases. This review will examine the present literature on mitochondrial-based treatments for neurodegenerative illnesses, including the reasoning behind these interventions, the progress made, and the remaining challenges.

## The pathogenesis of neurodegenerative diseases

The most common neurodegenerative disorders are Alzheimer’s disease and Parkinson’s disease (Lamptey et al. 2022). These disorders arise when nerve cells in the brain or peripheral nervous system gradually deteriorate in function and ultimately perish. Although existing medications may mitigate specific physical and cognitive symptoms, no therapies presently exist that can decelerate disease development or offer a cure. The likelihood of getting neurodegenerative illnesses markedly escalates with age. With the ongoing growth in life expectancy, the prevalence of neurodegenerative disorders among Americans is anticipated to climb in the forthcoming decades. Enhancing our comprehension of the etiologies of these diseases and formulating novel approaches for their treatment and prevention is imperative. Researchers recognize that genetic and environmental factors influence an individual’s susceptibility to neurodegenerative disorders. For instance, a person may carry a gene that predisposes them to Parkinson’s disease, but environmental exposures can influence the likelihood, timing, and severity of its onset.

**Fig. 4:**
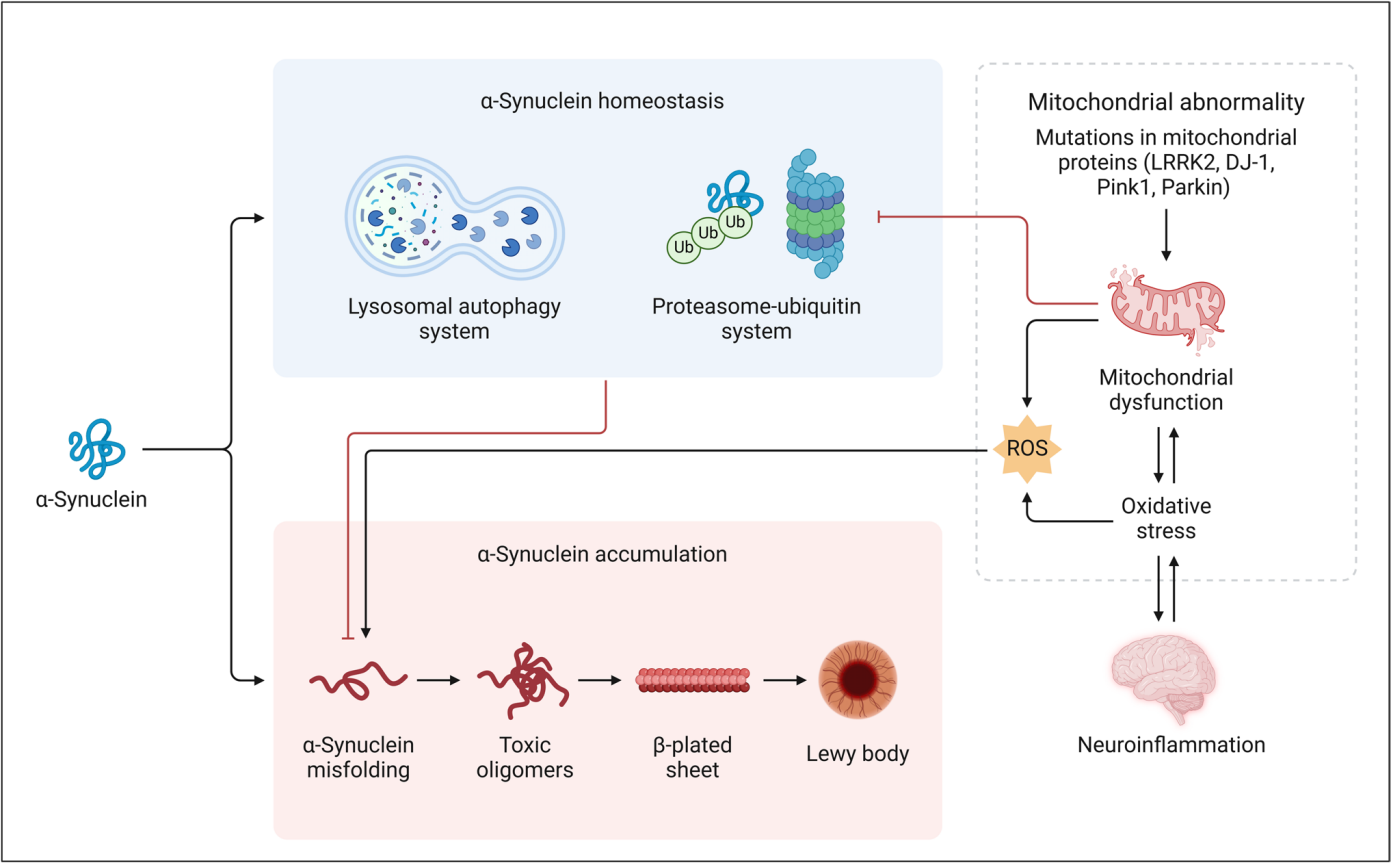
Schematic representation of α-synuclein homeostasis, accumulation, and pathological consequences. α-Synuclein is regulated by the lysosomal autophagy and proteasome-ubiquitin systems, maintaining its homeostasis (blue panel). Dysregulation of these systems leads to α-synuclein misfolding and the formation of toxic oligomers, β-plated sheets, and ultimately Lewy bodies (red panel). Mitochondrial abnormalities, including mutations in proteins such as LRRK2, DJ-1, Pink1, and Parkin, contribute to mitochondrial dysfunction and increased ROS production. This results in oxidative stress, neuroinflammation, and further exacerbation of α-synuclein toxicity (dotted panel). These interconnected pathways play a critical role in neurodegenerative diseases, particularly Parkinson’s disease

Neurodegenerative diseases are a group of chronic neuroinflammatory disorders that affect the brain parenchyma and structures of the brain. PD is recognized as one of the two predominant neurodegenerative disorders associated with aging, alongside Alzheimer’s disease. It is estimated that around 1–2% of individuals aged 65 and older are affected by this condition; however, PD may present at an earlier age as a result of genetic mutations, particular genetic predispositions, and/or exposure to environmental factors, medications, or physical injuries. Despite the availability of symptomatic medicines, no conclusive disease-modifying medications have been established. In the last two decades, progress in genetics has markedly improved our understanding of PD, with several newly discovered genes presenting prospective treatment targets. The initial gene associated with Parkinson’s disease (PD) was α-synuclein, with pathogenic mutations categorized as single-point mutations and substantial copy number changes, resulting in genomic duplications and triplications on chromosome 4q22.1 (Fig. 4) (Sastre et al. 2023). Clinically, an apparent gene dosage effect has been observed, with individuals carrying SNCA triplications showing earlier disease onset and more rapid progression. Although the exact thresholds for safely downregulating α-synuclein are still unknown, medical genetic insights and molecular and structural analyses of toxic alpha-synuclein aggregates, along with neuropathological evidence of alpha-synuclein accumulation in Lewy bodies, have supported the prevailing hypothesis that reducing alpha-synuclein levels may provide therapeutic advantages for PD and associated alpha-synucleinopathies (Sastre et al. 2023).

Moreover, the connection between the orexinergic system (Al-Kuraishy et al. 2020), brain liver X receptor (Alnaaim et al. 2024), ketogenic diets (Al-Kuraishy et al. 2024), and the amyloid precursor protein/amyloid beta axis (Al-Kuraishy et al. 2023) shows how different factors contribute to neurodegenerative and psychiatric disorders. Problems with orexin signaling are linked to sleep issues, cognitive decline, and mood disorders, making it a key factor in both psychiatric and neurodegenerative diseases. At the same time, the brain-specific liver X receptor plays a role in Parkinson’s disease by affecting lipid metabolism, reducing inflammation, and lowering oxidative stress, all of which are involved in the loss of neurons. On the metabolic side, ketogenic diets help protect the brain in Alzheimer’s disease by providing an alternative energy source, lowering oxidative stress, and improving neurotransmitter balance, which helps slow down memory loss. A significant issue in these conditions is the buildup of amyloid beta due to how amyloid precursor protein is processed. The amyloidogenic pathway leads to harmful amyloid deposits, while the non-amyloidogenic pathway may have protective effects. Finding ways to control this process, whether by changing metabolism through ketosis, influencing orexinergic activity, or using LXR activation, could help slow down neurodegeneration (Al-Kuraishy et al. 2020; Al-Kuraishy et al. 2023; Alnaaim et al. 2024).

## Mechanisms of mitochondrial dysfunction in neurodegeneration

Impaired oxidative phosphorylation is the source of many pathological diseases marked by decreased cellular energy supply. Oxidative phosphorylation, which occurs within the inner mitochondrial membrane, is an optimized workflow that connects the ETC to ATP formation. Electrons obtained from food oxidation go via a succession of protein complexes (I–IV) anchored in the mitochondrial inner membrane, eventually converting molecular oxygen to water. The energy released during this transfer is utilized to form a proton gradient, driving ATP synthase activity and creating ATP—the primary energy currency of the cell (Nicholls and Ferguson 2013). When the efficiency of oxidative phosphorylation is reduced, cells face an energy shortage, which can significantly impact their function and survival. Several reasons can cause decreased oxidative phosphorylation. Genetic flaws in mtDNA or nuclear-encoded ETC proteins can disrupt one or more complexes, decreasing electron flow and ATP production. Furthermore, mutations that impact mitochondrial DNA replication, repair, or stability can cause a buildup of dysfunctional mitochondria, increasing energy shortages. Environmental stresses, such as ischemia-reperfusion injury, exposure to chemicals, or chronic inflammation, can also damage mitochondria, reducing their ability to produce ATP efficiently (Wallace 2010).

Decreased oxidative phosphorylation efficiency causes cells to rely more heavily on less efficient energy-producing processes, such as glycolysis. While glycolysis can maintain baseline ATP generation, it frequently fails to compensate for oxidative phosphorylation capability loss fully. This alteration in energy metabolism can result in altered redox states and the buildup of intermediates and byproducts that worsen oxidative stress. Furthermore, persistent energy shortages affect ion homeostasis, disturb membrane potential maintenance, and increase sensitivity to apoptosis (Zorov et al. 2014). Decreased oxidative phosphorylation can seriously affect tissues with high energy needs, such as the brain, heart, and skeletal muscles. Neurological illnesses, particularly some types of dementia, have been related to mitochondrial malfunction and energy deficiencies. Similarly, impaired oxidative phosphorylation has been linked to heart failure, where low ATP availability impairs contractility and cardiac output. Age-related disorders typically display symptoms of mitochondrial malfunction and decreased oxidative phosphorylation, highlighting the crucial relevance of effective mitochondrial energy metabolism for sustaining cellular and tissue health (Bratic and Larsson 2013).

**Fig. 5:**
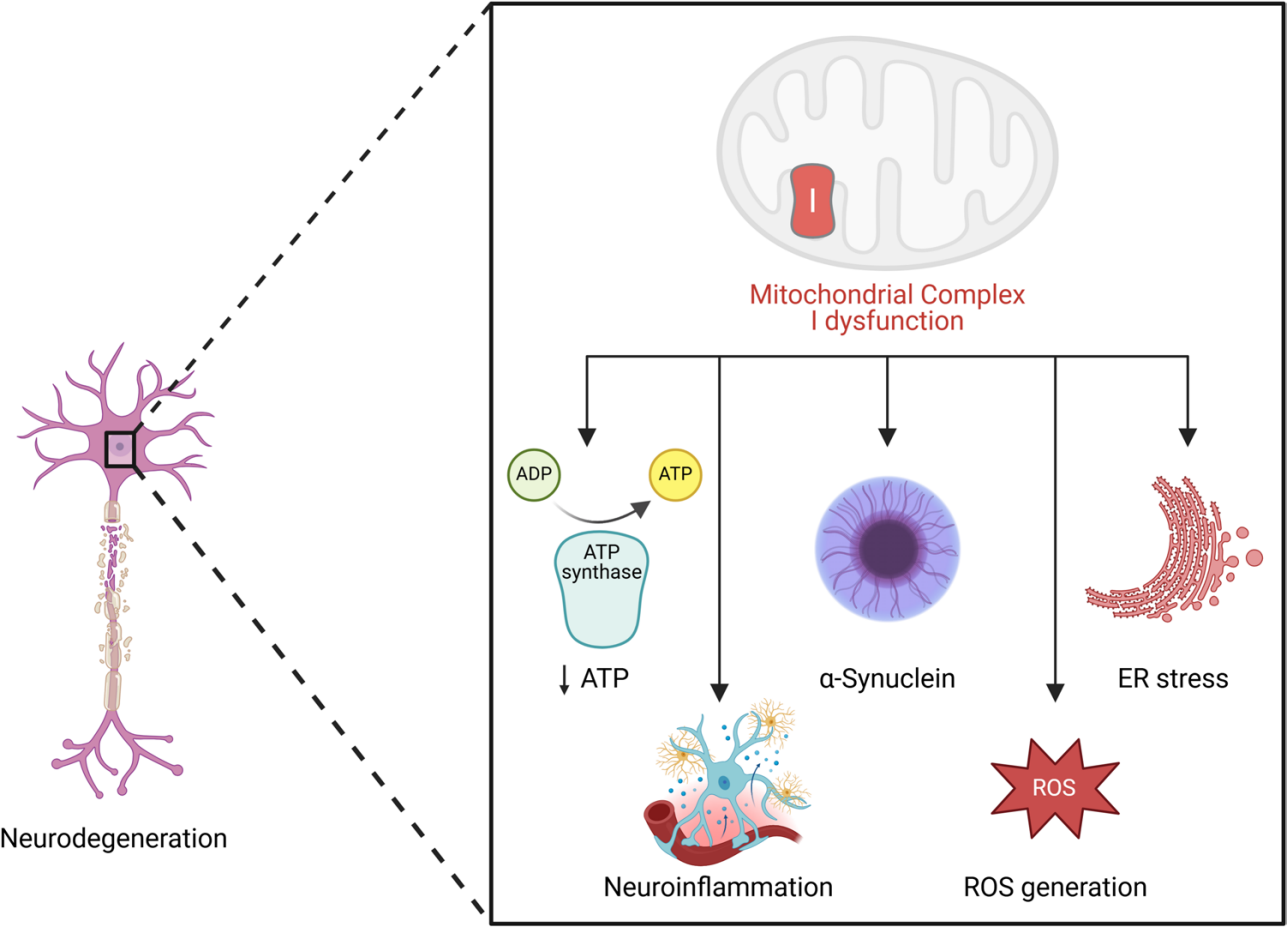
Mitochondrial complex I dysfunction and its role in neurodegeneration. The schematic illustrates how dysfunction in mitochondrial complex I leads to multiple pathological consequences contributing to neuronal degeneration. Impairment of complex I results in decreased ATP production due to reduced activity of ATP synthase, leading to energy deficits. Additionally, mitochondrial dysfunction is associated with increased α-synuclein aggregation, a hallmark of neurodegenerative diseases such as Parkinson’s disease. Furthermore, complex I impairment induces neuroinflammation, characterized by activation of inflammatory pathways in glial cells, exacerbating neuronal damage. Endoplasmic reticulum (ER) stress is another consequence of mitochondrial dysfunction, disrupting cellular protein homeostasis and further contributing to neuronal injury. Moreover, mitochondrial complex I dysfunction promotes the excessive generation of ROS, leading to oxidative stress and cellular damage. These interconnected pathological processes collectively drive neurodegeneration, highlighting the critical role of mitochondrial health in maintaining neuronal function

Furthermore, recent and novel research has highlighted the role of mitochondrial complex I (MCI) in neurodegeneration. There is mounting evidence that dysfunctional mitochondrial bioenergetics play an important role in the development of PD. The most harmful factor is a disruption in the electron transport chain or, more specifically, a disruption of the mitochondrial complex I (MCI) (Fig. 5). Because of their higher susceptibility to MCI damage, dopaminergic neurons endure oxidative stress and a compromise in ATP synthesis, leading to neurodegeneration and PD (Tryphena et al. 2023). Additionally, recent research highlights the pivotal role of histone modifications in regulating MQC, mainly through acetylation and methylation processes. Histone acetylation, driven by histone acetyltransferases (HATs), enhances gene expression linked to mitochondrial biogenesis, while histone deacetylases (HDACs) can repress these genes, consequently impairing mitochondrial function. Additionally, the modifications of histones such as H3K27ac and H4K12 have been associated with critical mitochondrial functions, influencing the expression of genes like PGC-1α, essential for mitochondrial biogenesis (Tryphena et al. 2023; Martins 2016; Pietrocola et al. 2015).

Epigenetic agents that target these histone modifications, such as HDAC inhibitors, have shown promise in preclinical studies, indicating their potential to enhance mitochondrial dynamics and function (Ambekar et al. 2021; Sanchez-Mut et al. 2014; Uittenbogaard and Chiaramello 2018; Wang et al. 2019). For instance, sirtuin activators, which promote histone deacetylation coupled with enhanced mitochondrial respiration, have emerged as a therapeutic strategy for addressing mitochondrial dysfunction, particularly in neurodegenerative diseases. Additionally, compounds like resveratrol and other natural histone-modifying agents are being investigated for their neuroprotective effects through modulation of histone acetylation patterns, suggesting a novel approach in the treatment of conditions marked by mitochondrial impairment (Cieslar-Pobuda et al. 2020; Gao and Zhang 2014; Hoglinger et al. 2003). In addition, several specific miRNAs have been identified as critical regulators of mitochondrial dysfunction, contributing to neurodegenerative diseases, particularly PD (Table 1).

**Table 1:** The role of specific miRNAs in mitochondrial quality control

MiRNA	Role in MQC
miR-7	Modulates expression of alpha-synuclein
miR-34b/c	Implicated in mitochondrial dysfunction
miR-103a-3p	Regulates Parkin, essential for mitochondrial quality control
miR-146a	Involved in neuroinflammatory processes
miR-494	Connected to DJ-1 protein levels for oxidative stress protection
miR-27a/b	Regulates PINK1, critical for mitochondrial maintenance
miR-181a	Correlates with mitochondrial function and neuronal health
miR-145-3p	Modulates DJ-1 levels for mitochondrial homeostasis

Also, mitochondria produce the ATP needed to control calcium levels and manage cell apoptosis. Mitochondria are always in structural remodeling through many processes of fission-fusion and mitophagy; all of this protects and ensures the continuous function of the mitochondria and cellular homeostasis. Any problem in these processes is combined with many diseases like neurological disorders, cancer, and cardiovascular disease (Table 2) (Kathiresan et al. 2024).

**Table 2:** Mitochondrial dysfunctional activities in AD, PD, and ALD

Disease	Mitochondrial dysfunction	Specific mutations in the mtDNA	Changes in mitochondrial dynamics	Mitophagy impairments
AD	Mitochondrial dysfunction is critical in neuronal degeneration, particularly in energy metabolism and oxidative stress.	Mutations in mitochondrial genes related to oxidative phosphorylation, like ND1 and ND4, have been linked to AD.	Decreased mitochondrial biogenesis, altered mitochondrial morphology, and impaired fusion-fission balance. Mitochondria become fragmented.	Impaired mitophagy leads to the accumulation of damaged mitochondria in neurons. Decreased Parkin and PINK1 activity.
PD	PD is characterized by mitochondrial dysfunction, which leads to reduced ATP production and increased oxidative stress, mainly affecting dopaminergic neurons.	Mutations in mtDNA, such as those in the ND6 gene, are linked to PD. Additionally, polymorphisms in mitochondrial genes like ATP6 have been implicated.	Impaired mitochondrial fusion, altered distribution of mitochondria, and increased mitochondrial fragmentation, especially in dopaminergic neurons.	Dysfunctional mitophagy due to impaired Parkin/PINK1 signaling pathways, resulting in the accumulation of damaged mitochondria.
ALS	Mitochondrial dysfunction is a central feature that contributes to motor neuron degeneration and cell death in ALS.	Mutations in ALS-associated genes (like SOD1, C9orf72) can also affect mitochondrial function. SOD1 mutations may alter mitochondrial activity.	Mitochondrial fragmentation, disrupted axonal transport, and altered calcium buffering. Mitochondria fail to undergo proper fission and fusion.	Impaired mitophagy, due to dysfunctions in the autophagic machinery, leads to the accumulation of damaged mitochondria. Defects in the PINK1/parkin pathway, exacerbating mitochondrial dysfunction.

### Mitochondrial fission

Fission of the mitochondria is separating one single mitochondrion into two new organelles. Fission is crucial because it sends the new mitochondria to the daughter cells during cell division. Allowing quality control of the cell and having the cellular energy needed to function the cell. Fission occurs by Drp1 and is linked to the mitochondrial outer membrane via adaptors such as the MFF. Once at the membrane, Drp1 produces oligomeric rings, which tighten and split the mitochondrion (Zerihun et al. 2023).

The disruption of fission can have lasting effects. High fission affects the mitochondria, lowering energy output and triggering apoptosis. In contrast, insufficient fission results in defective elongated mitochondria and the inability to correctly separate damaged parts. These aberrations are linked to diseases such as Alzheimer's dementia, where incorrect fission increases oxidative stress and brain injury (Wang et al. 2020).

### Mitochondria fusion

**Fig. 6:**
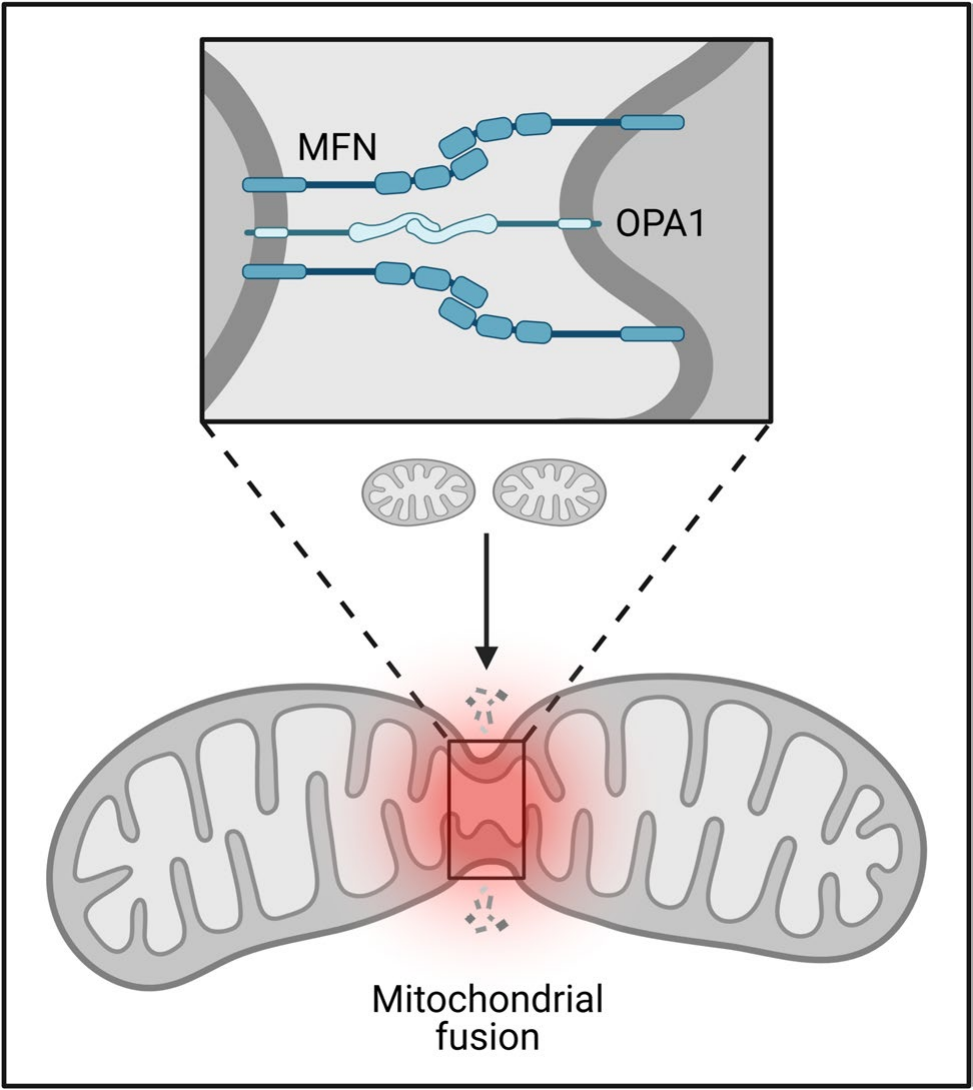
Schematic representation of mitochondrial fusion. The lower part of the figure illustrates the merging of two mitochondria into a single, larger mitochondrion. The inset highlights the molecular machinery involved in the fusion process, including MFN on the outer mitochondrial membrane and OPA1 on the inner mitochondrial membrane. These proteins mediate the fusion of mitochondrial membranes, essential for mitochondrial function, energy production, and cellular homeostasis

Fusion, or joining two mitochondria, permits mtDNA and proteins to combine (Fig. 6). This approach minimizes harm by diluting defective mtDNA and repairing mitochondrial membranes. On the outer membrane, mitofusins (Mfn1 and Mfn2) assist fusion, and on the inner membrane, ocular atrophy protein 1 (OPA1) assists fusion (Frey 2024). Impaired fusion weakens the mitochondrial network, decreasing the cell’s response to stress. This is especially harmful to neurons and heart cells, which rely on mitochondria for energy to function. In neurological disorders such as Charcot–Marie–Tooth disease and optic atrophy, mutations in Mfn2 and OPA1 have been linked to mitochondrial and cellular dysfunction (Li et al. 2023).

### Mitophagy

Mitophagy is the selective elimination of damaged mitochondria through autophagy, which is critical for sustaining mitochondrial integrity and minimizing the buildup of dysfunctional organelles. PTEN-induced putative kinase 1 (PINK1) tags damaged mitochondria and recruits them to the E3 ubiquitin ligase Parkin, where they are degraded by autophagosomes (Ge et al. 2020).

Mitophagy disruption results in the buildup of damaged mitochondria, which increases ROS production and causes cellular stress. This has been linked to Parkinson’s disease, in which mutations in PINK1 and Parkin impede mitophagy and cause neuronal degeneration. Similarly, poor mitophagy in various tissues contributes to metabolic problems, aging, and cancer progression (O’Callaghan et al. 2023).

### Mitochondrial fission, fusion, and mitophagy interplay

Fission, fusion, and mitophagy are all interrelated processes. For example, fission promotes mitophagy by segregating damaged mitochondria, whereas fusion preserves mitochondrial functioning and inhibits unnecessary mitophagy. The delicate balance of these systems is essential for safeguarding mitochondrial dynamics. Disruption of one component typically disturbs the others, leading to disease development (Preminger and Schuldiner 2024). Therapeutic approaches that target mitochondrial dynamics offer promise in treating illnesses associated with dysfunctional mitochondria. The current study focuses on manipulating fission and fusion proteins, boosting mitophagy, and using small molecules to restore mitochondrial function. Understanding these systems better would provide insight into their roles in health and illness, offering new avenues for intervention (Hong et al. 2024).

### Axonal transport

**Fig. 7:**
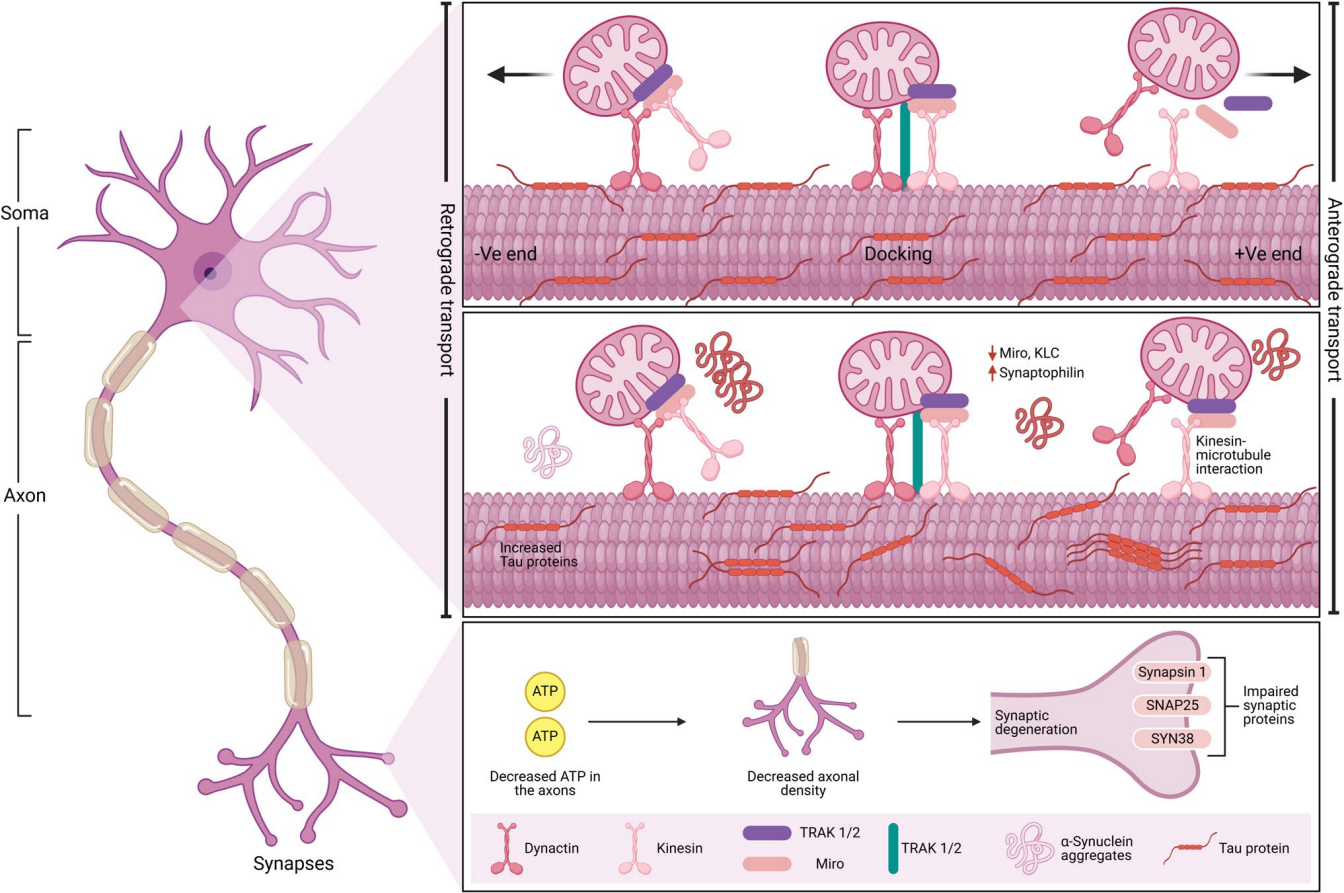
Overview of mitochondrial axonal transport in physiological and pathological conditions. The above panel represents mitochondrial transport in healthy neurons. Mitochondrial transport is facilitated by various motor and adaptor proteins that navigate along microtubules. The anterograde transport of mitochondria, directed toward axons, is mediated by kinesin, MIRO, and TRAK1/2, while dynein is responsible for retrograde transport. Additionally, syntaphilin functions as a docking protein, regulating mitochondrial positioning within the axon. The middle panel represents pathological disruptions in mitochondrial transport. In the presence of α-synuclein, MIRO and kinesin light chain (KLC) expression levels are reduced, whereas Tau and syntaphilin are upregulated. This pathological environment also leads to microtubule disintegration and impaired polymerization, hindering mitochondrial transport along axons. The bottom panel represents the consequences of impaired mitochondrial transport**.** Disruptions in mitochondrial transport result in insufficient mitochondria supply within axons, leading to an energy deficit, reduced axonal density, and compromised synaptic integrity. The dysfunction of synaptic proteins, including synapsin 1, SNAP25, and SYN38, ultimately contributes to synaptic degeneration

Axonal transport of mitochondria plays a crucial role in maintaining neuronal function. It is intricately linked to the pathogenesis of neurodegenerative diseases such as AD, PD, and HD (Fig. 7). Mitochondria are essential for calcium buffering, synaptic activity, and neuronal health (Lopes 2024; Venneman and Berghe 2024). The mitochondrial disruption can impair mitochondrial distribution, resulting in abnormal protein accumulation and autophagic defects, as observed in *Drosophila* models where axonal mitochondria are depleted (Lopes 2024). The dysregulation of mitochondrial dynamics, including impaired fission and fusion processes, further exacerbates oxidative stress and neurotoxicity, contributing to neuronal degeneration (Saxton and Hollenbeck 2012; Misgeld et al. 2007).

The precise transport and distribution of mitochondria are critical for maintaining local neuronal activities. Motor proteins, adaptor complexes, and signaling pathways tightly regulate mitochondrial transport in neurons (Table 3) (Hollenbeck and Saxton 2005; Millecamps and Julien 2013; Hung 2021). Dynein and kinesin motors drive bidirectional movement along microtubules, with kinesin-1 facilitating anterograde transport (toward synapses) and dynein mediating retrograde transport (toward the soma).

**Table 3:** Key regulators in axonal transport

Regulatory protein/mechanism	Role
Mitochondrial Rho GTPase (Miro1/2)	These proteins anchor mitochondria to motor proteins, responding to Ca^2+^ levels to regulate transport dynamics.
Milton TRAK adaptor protein	Act as a bridge between Miro and Kinesin motors, controlling cargo attachment and detachment
Post-translational modifications	Phosphorylation of Miro and Milton and ubiquitination pathways modulate mitochondrial transport efficiency in response to cellular stress.
Cytoskeletal integrity	Microtubule-associated proteins, such as tau, impact mitochondrial transport by stabilizing or obstructing transport pathways, particularly in disease states.

## Excessive ROS and oxidative stress

Reactive oxygen species (ROS) are chemically reactive molecules derived from oxygen. They are inherent byproducts of cellular metabolism, namely, within mitochondria, during oxidative phosphorylation. ROS plays key physiological roles in moderation, such as signaling and pathogen defense. An imbalance between reactive oxygen species (ROS) generation and the cell’s capacity to neutralize these molecules with antioxidants results in oxidative stress, negatively impacting cellular function and general health (Jomova et al. 2023). ROS are created both inside and outside. Endogenously, they are primarily produced by mitochondrial electron transport chain activity, peroxisomal metabolism, and enzymatic activities involving oxidases and oxygenases. ROS is mainly generated in mitochondria, particularly during electron leakage from respiratory chain complexes I and III. Exogenous sources include UV radiation, environmental contaminants, tobacco smoke, and exposure to xenobiotics and heavy metals (Aranda-Rivera et al. 2022). Excessive ROS can interact with macromolecules in cells, causing oxidative damage to lipids, proteins, and DNA. Lipid peroxidation impairs membrane integrity, influencing cell signaling and organelle function. Protein oxidation causes structural changes, loss of enzyme activity, and aggregation, which disrupts cellular operations. DNA damage consists of strand breaks, base alterations, and cross-linking, which can cause mutations, genomic instability, and cell death if not repaired (Juan et al. 2021).

Oxidative stress is a key factor in the pathophysiology of many illnesses. ROS increases neuronal damage and promotes apoptosis in neurodegenerative diseases such as AD, PD, and ALS. Excessive ROS in cardiovascular disorders causes endothelial dysfunction, atherosclerosis, and myocardial damage by disrupting nitric oxide signaling and increasing inflammation (Juan et al. 2021). Cancer progression has also been linked to oxidative stress. While modest amounts of ROS might enhance cell proliferation and survival, prolonged oxidative stress can cause DNA mutations and genomic instability, resulting in oncogenesis. Furthermore, in metabolic disorders such as diabetes, high glucose levels promote ROS formation, injuring pancreatic beta cells and aggravating insulin resistance (Aranda-Rivera et al. 2022). Mitochondria are essential for energy production and cellular homeostasis; nevertheless, their failure is increasingly associated with the etiology of neurodegenerative disorders due to oxidative stress. Recent advances in mitochondrial-based therapies focus on MTAs as therapeutic strategies to counteract oxidative damage. The mechanisms by which ROS and RNS contribute to cellular injury emphasize the importance of maintaining redox homeostasis within mitochondrial environments. Various antioxidant approaches, both natural and synthetic, are classified into enzymatic and non-enzymatic categories, and nontargeted and mitochondria-targeted antioxidants are distinguished.

## Therapeutic strategies targeting mitochondrial dysfunction

### Mitochondria-targeted antioxidants (MTAs)

Mitochondria are essential organelles in all eukaryotic cells, utilizing around 98% of the body’s oxygen supply (Zong et al. 2024). Mitochondria generate energy through oxidative phosphorylation, using oxygen efficiently, a process enabled by the protein complexes of the electron transport chain (Choi et al. 2024). Under normal physiological conditions, approximately 3–5% of the oxygen within mitochondrial ETC complexes I and III may experience slippage, resulting in the formation of superoxide radicals (O₂•^−^), which are highly reactive and unstable molecules that can further contribute to the generation of additional ROS (Ore et al. 2024; Bhardwaj et al. 2022).

Mitochondria employ antioxidant defense mechanisms essential for cellular repair to mitigate elevated levels of free radicals. The enzyme superoxide dismutase swiftly transforms superoxide (O₂•^−^) into the less reactive hydrogen peroxide (H₂O₂), which is subsequently decomposed into water by catalase or neutralized by the peroxiredoxin and thioredoxin enzyme systems. Superoxide (O₂•^−^) can generate hydroxyl (•OH) radicals by the Fenton reaction, which may subsequently produce peroxynitrite (ONOO−) and other reactive nitrogen species, including nitrogen dioxide and peroxynitrous acid. Mitochondria typically uphold redox equilibrium via antioxidant mechanisms that mitigate reactive oxygen and nitrogen species; however, an overabundance of ROS and RNS can induce oxidative harm to cellular constituents, such as proteins, lipids, nucleic acids, and the extracellular matrix (Pal et al. 2024). Key markers of peroxynitrite-induced oxidative stress include oxidized protein adducts, such as 3-nitrotyrosine, protein carbonylation, and the lipid peroxidation product 4-hydroxynonenal (4-HNE). Furthermore, ROS and RNS can damage both nuclear and mitochondrial DNA, affecting gene expression and cellular function. Excessive production of ROS and RNS may also impair electron transport chain subunits, thereby sustaining a cycle of mitochondrial dysfunction. In light of these issues, several clinical trials are investigating therapeutic approaches to alleviate mitochondrial dysfunction in various neurodegenerative diseases (Mohamed et al. 2023; Halling and Pilegaard 2020) (Table 4).

**Table 4:** Current clinical trials targeting mitochondrial dysfunction in neurodegenerative diseases

Clinical trials registered number on ClinicalTrials.gov	Treatment	Phase	Number of patients
Alzheimer’s disease			
NCT05617508	NR	-	80
NCT04430517		I	50
NCT05040321	MIB-626	I/II	50
NCT04842552	Hydralazine	III	424
NCT05591027	*Centella asiatica* product	I	48
NCT04740580	Glycine, NAC		52
NCT05081219	Insulin, empagliflozin	II	60
NCT04018092	Photobiomodulation		168
NCT04784416			125
Amyotrophic lateral sclerosis			
NCT04820478	Beta-hydroxybutyrate ester	-	76
NCT04244630	Antioxidant supplements	II	60
Huntington’s disease			
NCT01502046	THC and CBD	II	25
Parkinson’s disease			
NCT04477161	Oral ketone esters	-	10
NCT03840005	Ursodeoxycholic acid	II	31
NCT04044131	Serine, L-carnitine, NAC, NR	I	120
NCT05214287	Intermittent hypoxia therapy	I, II	29
NCT04768023	Vitamin D3	-	50

**Fig. 8:**
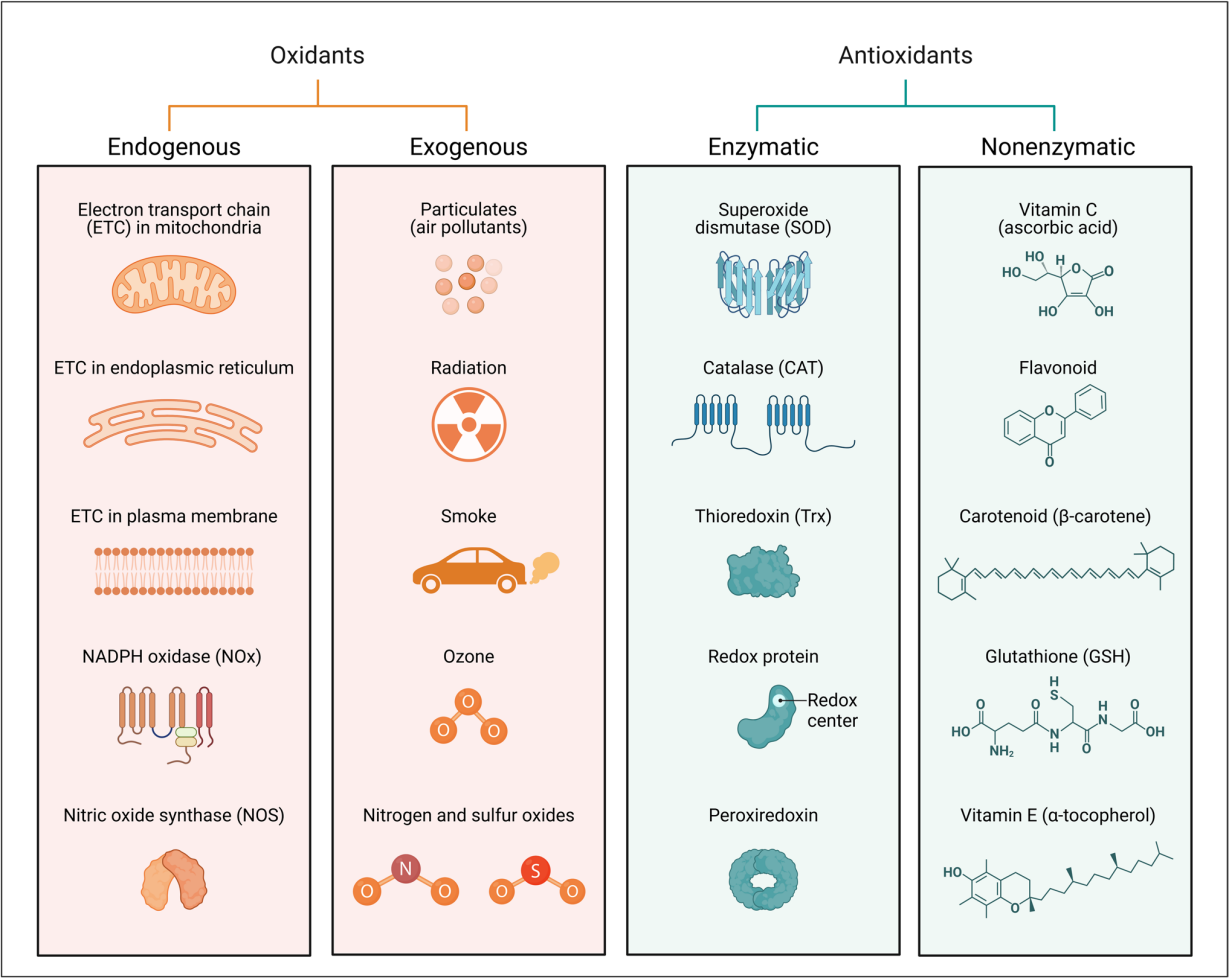
Overview of oxidants and antioxidants, classified into endogenous and exogenous sources, and enzymatic and nonenzymatic antioxidant defenses. The oxidants panel highlights the sources of reactive oxygen species (ROS) and other reactive species: Endogenous oxidants (left column) are primarily generated as byproducts of cellular metabolism, including the electron transport chain (ETC) in mitochondria, endoplasmic reticulum, and plasma membrane. Additional endogenous sources include NADPH oxidase (NOx), an enzyme complex involved in ROS production, and nitric oxide synthase (NOS), which contributes to reactive nitrogen species (RNS). Exogenous oxidants (center column) include environmental factors such as particulates from air pollutants, ionizing radiation, cigarette smoke, ozone, and chemical compounds like nitrogen and sulfur oxides, all contributing to oxidative stress. Enzymatic antioxidants (right column, blue box) include enzymes such as superoxide dismutase, which catalyzes the dismutation of superoxide radicals into oxygen and hydrogen peroxide; catalase, which decomposes hydrogen peroxide into water and oxygen; thioredoxin (Trx), which maintains a reduced intracellular environment; redox proteins involved in electron transfer; and peroxiredoxin, which reduces peroxides. Nonenzymatic antioxidants (right column, green box) include small molecules like vitamin C (ascorbic acid), which directly neutralizes free radicals; flavonoids, plant-derived polyphenols with antioxidant properties; carotenoids like β-carotene, which scavenge singlet oxygen; glutathione (GSH), a tripeptide involved in the detoxification of hydrogen peroxide and lipid peroxides; and vitamin E (α-tocopherol), a lipid-soluble antioxidant protecting cellular membranes from oxidative damage

Antioxidants mitigate cellular oxidative damage by neutralizing or scavenging free radicals, as highlighted in Table 5. Antioxidant therapy has gained attention as an innovative strategy for addressing neurodegenerative disorders, where oxidative stress significantly contributes to disease onset and progression (Fig. 8). Brain antioxidant levels can be restored via two primary mechanisms, offering potential therapeutic pathways to curtail free-radical production and oxidative stress. This restoration subsequently enhances redox homeostasis following brain-related disorders. In pathological neural tissues, antioxidants may modulate redox processes by detoxifying or scavenging excessive ROS and RNS through natural or synthetic methods, inhibiting free radical overproduction, and affecting signaling pathways that promote endogenous antioxidant synthesis, thus preserving redox equilibrium (Modi et al. 2024).

**Table 5:** Classification of antioxidants according to their pharmacological properties and subcellular targets

Antioxidants				
Natural ROS-RNS scavengers		Synthetic ROS-RNS scavengers and detoxifiers		
Enzymatic	Non-enzymatic	Cytosolic	Mitochondria-targeted	Glutathione-precursors
SOD CAT GPx Trx Prx TrxR NADPH	Vitamins A, C, and E Cu, Zn, Se, and Mg Minerals Flavinoids Polyphenols CoQ10 Glutathione NADPH CytC	Allin Allicin MnTBAP Cysteine Tiron	Carbon-PTIO MitoVit-E MitoQ SKQ1 Edaravone Mito TEMPOL Elamipretide Nano-CeO2 Metallopolypherons PBN	NAC NACA D-NAC SAMe

Table 5 categorizes antioxidants into synthetic and natural ROS-RNS scavengers and detoxifiers. Synthetic antioxidants are categorized into non-targeted cytosolic, mitochondria-targeted, and glutathione precursors. Natural antioxidants are categorized into enzymatic and non-enzymatic types.

Research emphasizes the development of targeted mitochondria antioxidants (TMAs) instead of nontargeted antioxidants (NAs). Excessive use or improper application of antioxidants may inhibit ROS generation, leading to compensatory activation of MAPK pathways, compromising the endogenous antioxidant system, and affecting normal cellular development (Jiang et al. 2020). Another critical issue lies in the absorption and metabolism of non-targeted antioxidants across various organs. Antioxidants can be conjugated with carriers such as lipophilic cations, liposomes, or peptides to boost therapeutic efficacy, permitting targeted distribution to mitochondria. This method facilitates significant accumulation within cells and mitochondria, protecting against oxidative damage via various mechanisms. Effective antioxidants must exhibit high bioavailability, facilitating swift entry into circulation through intestinal absorption or intravenous administration. MTAs are especially beneficial as they localize within mitochondria and protect specific tissues from oxidative stress, including the brain, liver, kidneys, muscles, ears, and heart. In the last 10 years, substantial advancements have been achieved in developing MTAs, encouraging findings that will be discussed in detail (Jiang et al. 2020; Bishop et al. 2019; Chen et al. 2022).

### Ethical and regulatory issues in mitochondrial-based therapie

Mitochondrial replacement therapy (MRT) presents significant ethical and regulatory challenges, particularly concerning genetic modification and the implications of involving a third genetic contributor (Dimond 2023; Noohi et al. 2022). In the USA, MRT is largely unregulated, with prohibitions stemming from political debates surrounding reproductive rights and fetal personhood, which complicates access to funding and research opportunities (Hirano et al. 2021). Globally, the UK and Australia have established regulatory frameworks, yet ethical concerns persist regarding the identity implications for children born through MRT and the potential risks to mitochondrial donors (Newson 2022). The technology raises questions about parental rights, the definition of family, and the societal implications of germline modifications, necessitating a careful evaluation of the ethical landscape as more jurisdictions consider MRT's clinical applications (Pike 2022; Dolitsky and Sauer 2019).

## Enhancing mitochondrial biogenesis

### PGC-1α activators

The transcriptional coactivator PGC-1 alpha significantly enhances mitochondrial biogenesis, ensuring cells’ energy metabolism and proper function. It promotes mitochondrial replication and function by activating transcription factors, including NRF1, NRF2, and TFAM (Fontecha-Barriuso et al. 2020; Shelbayeh et al. 2023). Upon activation, PGC-1α translocates to the nucleus, stimulating the transcription of nuclear-encoded mitochondrial genes, increasing the synthesis of mitochondrial proteins and the replication of mitochondrial DNA mtDNA. Various activators, including AMPK and SIRT1, modulate PGC-1α activity through phosphorylation and deacetylation mechanisms, respectively (Higashida et al. 2013; Halling et al., 2020). These regulatory pathways are essential for adapting to metabolic demands during stressors like exercise, fasting, or hypoxia (Gureev et al. 2019). Additionally, PGC-1α is involved in mitochondrial quality control processes such as mitophagy and dynamics, ensuring the maintenance of healthy mitochondria. The therapeutic potential of PGC-1α activators in combating metabolic disorders and age-related decline highlights its significance in biomedical research (Chen et al. 2022; Dabrowska et al. 2015). Overall, targeting PGC-1α and its regulatory network is promising for enhancing mitochondrial biogenesis and improving metabolic health (Qian et al. 2024; Zhang et al. 2022).

### Exercise mimetics

Exercise mimetics are compounds that can replicate the beneficial effects of physical exercise, particularly in enhancing mitochondrial biogenesis. Mitochondrial biogenesis maintains cellular energy balance and overall metabolic health (Craig et al. 2015). These mimetics activate similar signaling pathways as exercise, notably those involving AMP-activated protein kinase (AMPK) and PGC-1α, key mitochondrial function and adaptation regulators. During exercise, increased AMP/ATP ratios activate AMPK, which subsequently phosphorylates PGC-1α, promoting its translocation to the nucleus and initiating the transcription of genes responsible for mitochondrial biogenesis (Hood et al. 2011). Research has identified various bioactive compounds, such as resveratrol, berberine, and curcumin, that can stimulate these pathways without physical activity (Jun et al. 2023). These compounds enhance oxidative capacity by increasing mitochondrial mass and enzyme activity, thus improving energy metabolism in skeletal muscle (Roberts and Markby 2021; Bishop et al., 2018; David et al. 2009; Johri and Beal 2012a, b). Furthermore, exercise mimetics can help mitigate age-related decline in mitochondrial function and combat metabolic disorders by promoting healthier muscle tissue. The ongoing investigation into these compounds holds promise for developing therapeutic strategies to harness the benefits of exercise for individuals unable to engage in traditional physical activity (Lilienthal et al. 2020; Pham et al. 2019).

Recent research has explored the combined impact of exercise and F-actin-mediated endocytosis on mitochondrial transplantation in experimental models of PD. A study by Jain et al. investigated the effects of transplanting mitochondria isolated from exercised mice into a PD mouse model. The findings revealed that exercise-conditioned mitochondria exhibited higher respiratory capacities than sedentary mice. When these enhanced mitochondria were transplanted into PD mice, there was a significant improvement in mitochondrial biogenesis and increased mitochondrial subunits, reducing PD pathology (Jain et al. 2023a, b).

### Modulating mitophagy

Regulating mitophagy is essential for improving mitochondrial biogenesis, as these processes collaboratively sustain mitochondrial health and functionality. Mitophagy is the selective destruction of impaired or defective mitochondria (Palikaras et al. 2015) and is pivotal for cellular homeostasis and energy metabolism (Hurtado and Schnellmann 2024). The balance between mitochondrial biogenesis and mitophagy ensures that new, functional mitochondria are produced while eliminating impaired ones. Key regulators of this interplay include PGC-1α, which promotes mitochondrial biogenesis, and various mitophagy receptors such as Parkin and PINK1, which tag damaged mitochondria for degradation (Borbély et al. 2022). The activation of mitophagy can augment mitochondrial biogenesis by inhibiting the buildup of defective mitochondria, which may obstruct cellular energy generation and elevate oxidative stress. For instance, during periods of metabolic stress or exercise, enhanced mitophagy clears damaged mitochondria and stimulates the synthesis of new mitochondria through signaling pathways involving AMPK and SIRT1 (Chen et al. 2022). Cells need to respond quickly and adequately to changes in energy needs. New research shows that drugs that target mitophagy can effectively increase mitochondrial biogenesis. This could lead to new ways of treating age-related diseases and metabolic disorders marked by mitochondrial dysfunction (Eliason et al. 2020).

### PINK1/Parkin pathway drugs

**Fig. 9:**
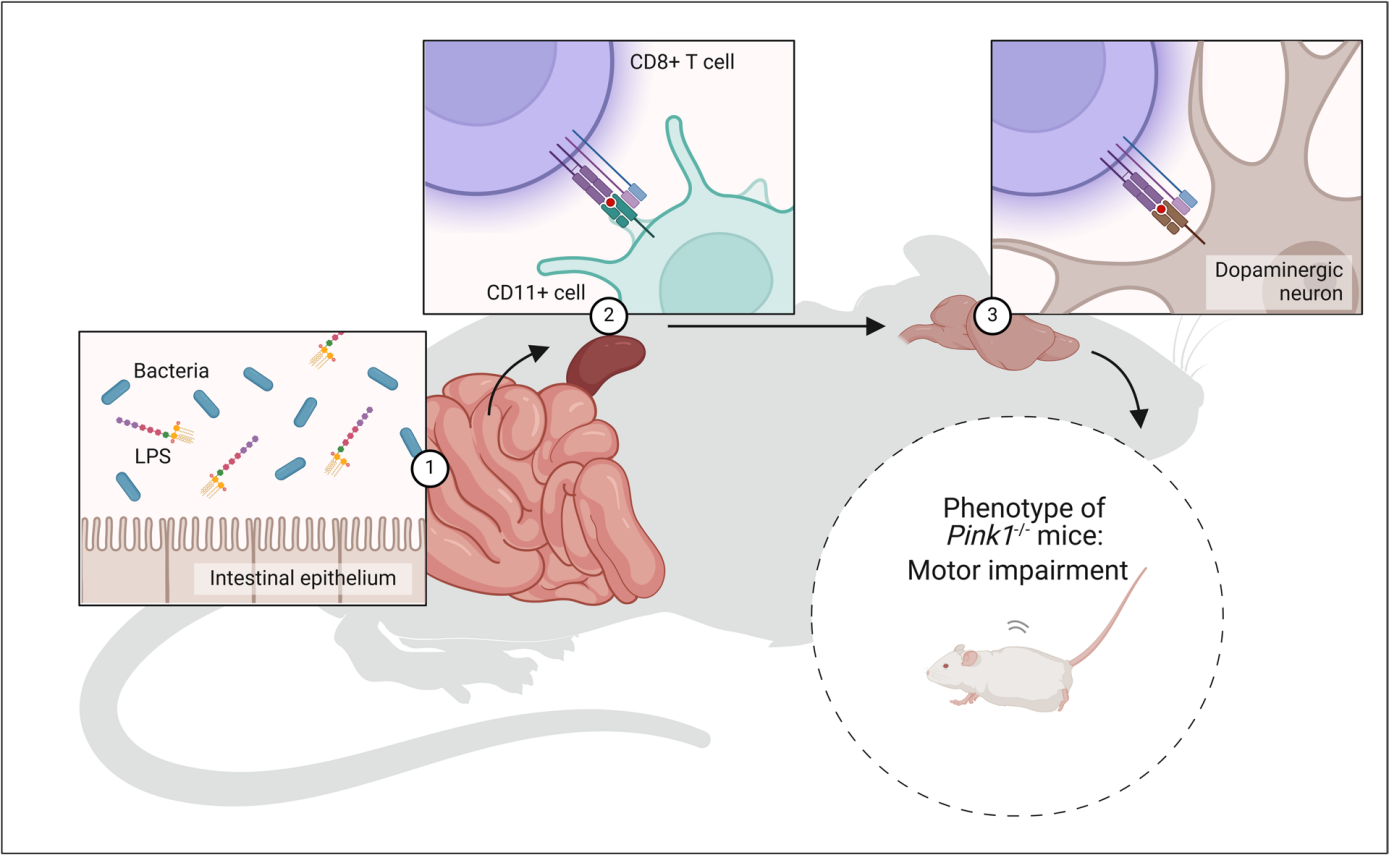
Mechanistic insights into gut-brain interactions in PINK1-deficient (Pink1^−^/^−^) mice, highlighting the role of immune responses and motor impairments. (1) Gut-derived immune activation: The intestinal epithelium is exposed to bacterial components, such as LPS, derived from the gut microbiota. These bacterial products breach the epithelial barrier and activate immune cells, including CD11⁺ cells, in the gut-associated lymphoid tissue. (2) Immune cell migration: CD11⁺ cells, upon activation, interact with CD8⁺ T cells, leading to systemic immune activation. This facilitates the trafficking of immune cells and pro-inflammatory mediators through systemic circulation, impacting distal organs, including the brain. (3) Impact on the brain and motor phenotype: The activated immune cells and inflammatory mediators infiltrate the brain, targeting dopaminergic neurons, particularly susceptible to immune-mediated damage in Pink1^−^/^−^ mice. This contributes to neuronal dysfunction and loss, manifesting as motor impairments, a characteristic phenotype of PINK1-deficient mice

The PINK1/Parkin pathway is essential for improving mitochondrial biogenesis because it helps with mitophagy, which removes damaged mitochondria (Yu et al. 2011). This mechanism is triggered under instances of mitochondrial stress, where research has shown that PINK1 concentrates on the outer mitochondrial membrane (known as OMM) of dysfunctional mitochondria in intestinal disease models (Fig. 9) (Zhang et al. 2022). Once stabilized, PINK1 recruits Parkin, an E3 ubiquitin ligase, which ubiquitinates OMM proteins, marking them for autophagic degradation (Ashrafi and Schwarz 2012). This process removes impaired mitochondria and stimulates the synthesis of new mitochondria, thereby promoting mitochondrial biogenesis (Ivankovic et al. 2016). Various pharmacological agents targeting this pathway have shown promise in enhancing mitochondrial function and biogenesis. For example, compounds that induce mitochondrial depolarization can activate PINK1/Parkin signaling, leading to increased mitochondrial turnover and improved cellular energy metabolism. Additionally, studies have demonstrated that drugs like BL-918 can enhance the PINK1/Parkin pathway, resulting in beneficial effects on mitochondrial dynamics and function. By modulating this pathway, it is possible to develop therapeutic strategies aimed at improving mitochondrial health in conditions such as neurodegenerative diseases and metabolic disorders where mitochondrial dysfunction is prevalent (Ivankovic et al. 2016).

Additionally, reduced levels of sphingosine-1-phosphate receptor 1 (S1PR1) are associated with impaired mitophagy, a critical process for mitochondrial quality control. The compound FTY720 (fingolimod) has been shown to enhance mitophagy through the S1PR1-Akt-BNIP3-PINK1-Parkin signaling axis (Pinjala et al. 2024). Additionally, FTY720 activates Parkin via the S1PR1-Akt pathway, further promoting mitophagy. These mechanisms suggest that targeting S1PR1 with modulators like FTY720 may offer neuroprotective benefits in PD by restoring mitophagy and improving mitochondrial function (Rajan et al. 2024)

## Mitochondrial treatments

### Mitochondrial transplantation

**Fig. 10:**
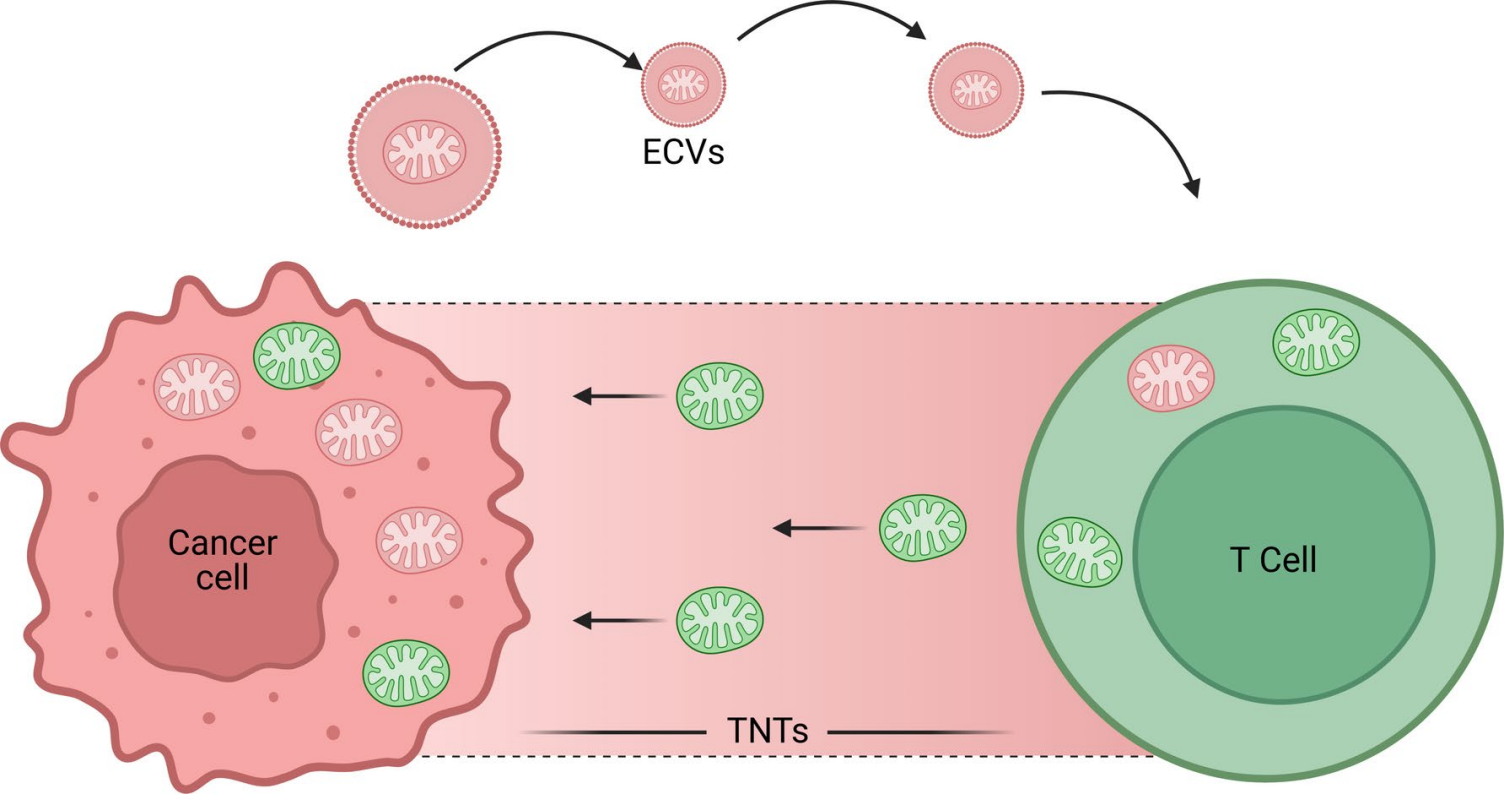
Schematic representation of mitochondrial transfer between a cancer cell and a T cell. The figure illustrates two mechanisms of mitochondrial transfer: extracellular vesicles (ECVs) and tunneling nanotubes (TNTs). The cancer cell (pink) donates mitochondria (green) to the T cell (green), which also contains its mitochondria (some depicted in pink to indicate possible dysfunctional mitochondria). ECVs mediate mitochondrial transfer through vesicle secretion, while TNTs facilitate direct cell-to-cell mitochondrial exchange. This process may influence T cell function in the tumor microenvironment

Mitochondrial transplantation techniques are emerging as innovative therapeutic approaches to restore mitochondrial function in various diseases characterized by mitochondrial dysfunction (Jain et al. 2023a, b; Gollihue et al. 2018; Morra et al., 2023). These techniques involve the transfer of healthy mitochondria into cells or tissues that exhibit impaired mitochondrial activity, thereby enhancing cellular energy production and reducing oxidative stress (Fig. 10) (McCully et al. 2023; Jain et al. 2023a, b). Several methods have been developed for mitochondrial transplantation, including direct microinjection, cell-mediated transfer, and systemic delivery (Antentor et al., 2024). Direct microinjection involves the precise introduction of isolated mitochondria into target cells, which has been shown to improve cell viability and function in models of ischemia-reperfusion injury.

**Fig. 11:**
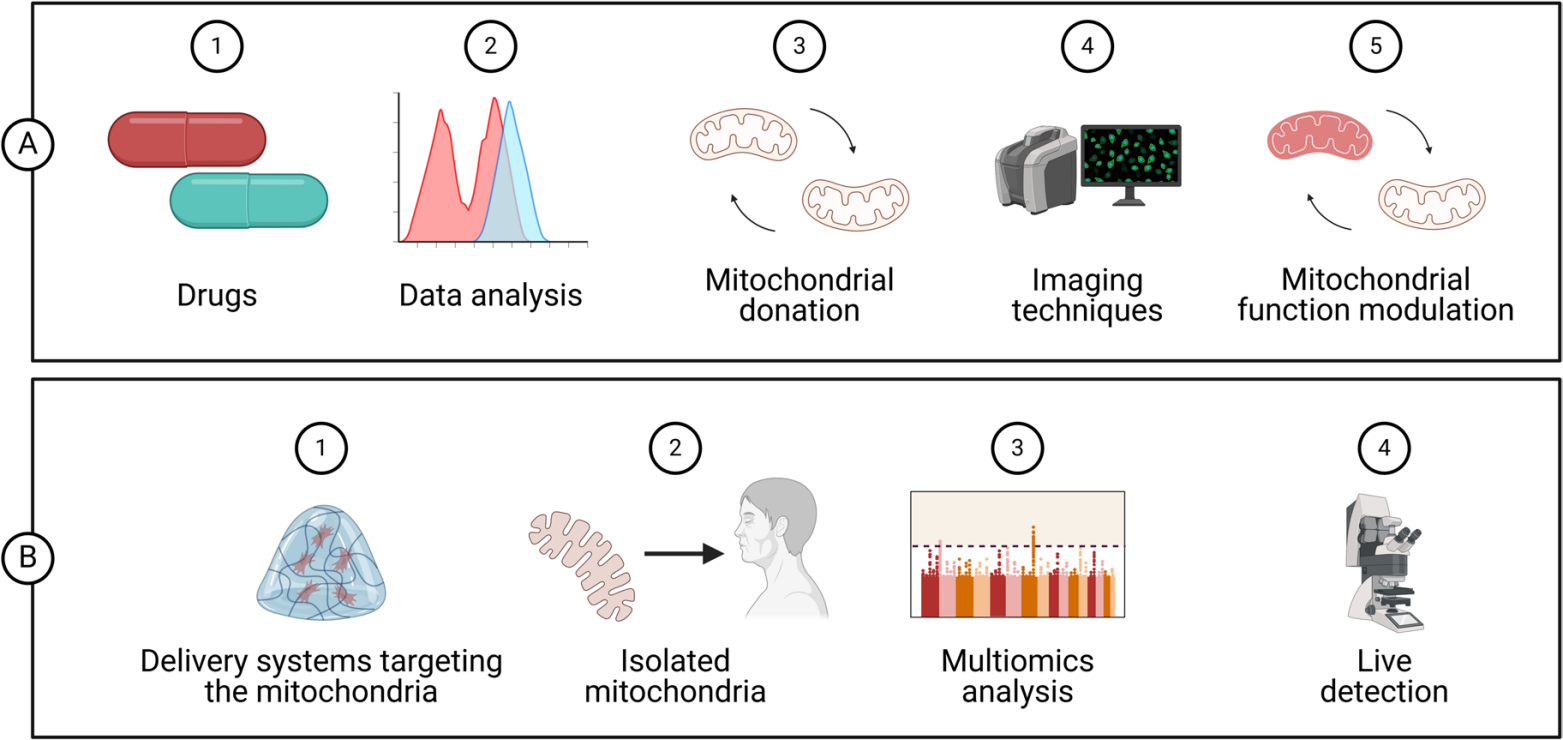
Regulatory strategies and therapeutic methods driven by mitochondria offer considerable promise in regenerative medicine. Approaches like mitochondrial transfer, advanced imaging tools, data analysis, and the functional modification of mitochondria are becoming viable solutions for treating mitochondrial dysfunction. Future developments are likely to concentrate on mitochondrial-targeted drug delivery systems, the creation of synthetic or isolated mitochondria for regenerative treatments, the use of advanced imaging and omics technologies for in-depth mitochondrial analysis, and the targeted, precise manipulation of mitochondrial function at the cellular level and within specific timeframes

Cell-mediated transfer utilizes donor cells, such as mesenchymal stem cells, which secrete vesicles containing mitochondria that can be taken up by recipient cells, facilitating mitochondrial exchange and enhancing bioenergetics (Fig. 11) (Halling et al., 2020). Systemic delivery methods allow for a broader distribution of mitochondria throughout the body, potentially benefiting multiple organs simultaneously. Recent studies indicate that mitochondrial transplantation can lead to significant improvements in conditions such as heart disease (Hassanpour et al. 2025), stroke (Huang et al. 2023), and neurodegenerative disorders (Bustamante-Barrientos et al. 2023; Riou et al. 2025). However, challenges remain regarding the optimal techniques for isolation, delivery, and integration of transplanted mitochondria into host cells. Continued research is essential to refine these methods and fully realize their therapeutic potential (Zhang et al. 2022).

### Challenges and limitations in mitochondrial transplantation

Mitochondrial transplantation presents several challenges and limitations that must be addressed before it can be widely adopted as a clinical therapy. One significant challenge is isolating and preserving viable mitochondria, which is crucial for successful transplantation (Riou et al. 2024; Walker et al. 2024). Current efforts focus on refining isolation techniques and cryopreservation methods to ensure the mitochondria remain functional until transplanted. Additionally, the source of mitochondria is critical, as metabolic compatibility between donor and recipient mitochondria is essential for effective treatment. Research indicates that mitochondria from different species can be used without significant immune response, but metabolic compatibility remains challenging. In acute clinical settings, such as during treatment for ischemic stroke, logistical challenges arise due to limited tissue samples and the constrained time available for isolation and transplantation. Furthermore, the potential for immune responses and rejection of donor mitochondria necessitates using autologous mitochondria, which limits the scope of MT applications (Bodenstein et al. 2024). Developing biomaterials to enhance mitochondrial uptake and dynamic models to mimic the recipient tissue environment are also areas of ongoing research to overcome these limitations (Wang et al. 2024).

In conditions like ischemia and stroke, one significant hurdle is the rapid deactivation of transplanted mitochondria and low transfer efficiency, which can hinder therapeutic outcomes in regions such as the cerebral cortex and spinal cord (Wei et al. 2025). Additionally, logistical issues arise during acute clinical settings, such as limited tissue samples and the constrained time for mitochondrial isolation and transplantation, which are critical for effective treatment during ischemic events (Walker et al. 2024). Furthermore, the mechanisms governing the fate of transplanted mitochondria remain poorly understood, complicating the optimization of transplantation protocols (Xie et al. 2021). Lastly, while metabolic compatibility between donor and recipient mitochondria is essential for enhancing cellular bioenergetics, competition among mitochondria of varying functions can affect the overall efficacy of the treatment. These factors collectively underscore the complexities of advancing mitochondrial transplantation as a viable therapeutic strategy in neurological disorders (Riou et al. 2024).

## Gene therapies

### CRISPR/Cas9

**Fig. 12:**
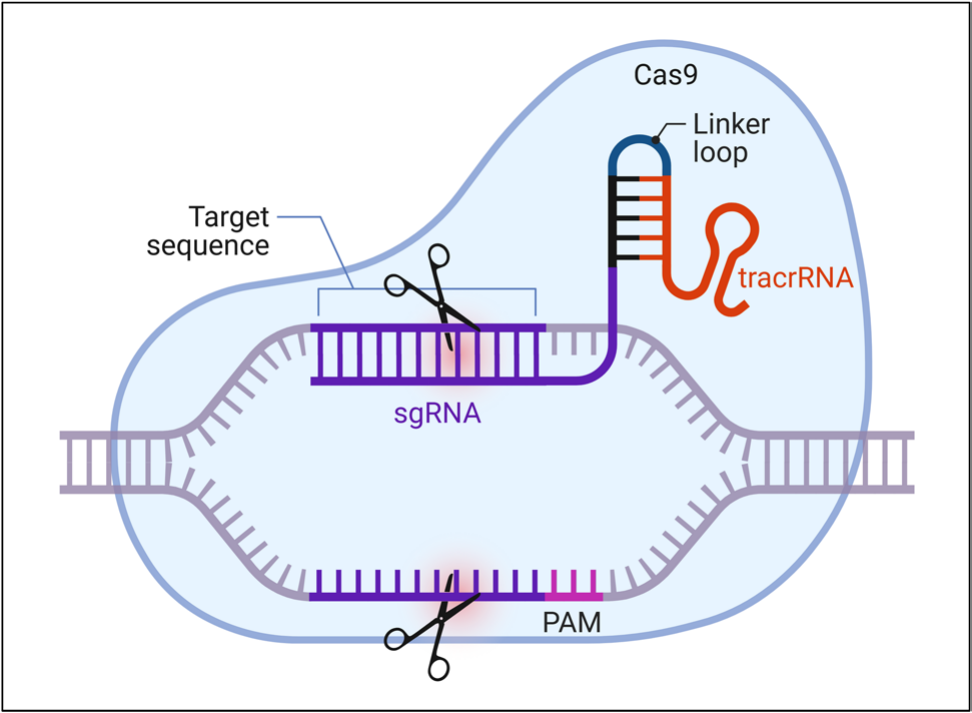
CRISPR/Cas9 gene editing tool components

CRISPR/Cas9 is an advanced genome-editing technology that improves the ability to delete or modify genetic material within living cells (Sahel et al. 2023). The acronym stands for clustered, regularly interspaced short palindromic repeats. The CRISPR/Cas9 system consists of the Cas9 endonuclease, which induces double-strand breaks (DSBs) in DNA at targeted locations, and a small guide RNA that directs Cas9 to the specific gene for modification (Fig. 12). This gene-editing technique has the potential to restore the function of essential biological pathways, which may be involved in PD (Sahel et al. 2023).

Moreover, CRISPR-Cas9 technology could prevent the transmission of hereditary PD by altering the DNA of germline cells and targeting gene mutations that lead to localized disease symptoms. Studies have found a link between mutations in the α-synuclein (SNCA) gene and neurological issues in the substantia nigra of PD patients. SNCA was the first gene associated with PD. Researchers have suggested that CRISPR-dCas9 could decrease SNCA expression levels, potentially offering therapeutic benefits for PD. This methodology allows for various genetic modifications, including the deletion of extensive nucleotide sequences, homologous recombination, insertion/deletion point mutations, and transcriptional alterations of specific genetic components. It has also been applied to address mitochondrial dysfunction associated with PD and neuronal survival. The CRISPR/Cas9 technique has illuminated critical factors involved in the pathogenesis of PD (Sahel et al. 2023). It aids in examining cellular elements that influence the susceptibility of cellular processes in this condition. The potential of CRISPR technology for PD treatment is promising, particularly in correcting mitochondrial mutations (Table 6). For instance, specific mutations in the MT-ND1 and MT-ND2 genes have been associated with Leigh syndrome, a severe neurodegenerative condition characterized by progressive loss of mental and movement abilities. Recent advancements in gene-editing technologies, particularly CRISPR, offer promising avenues for correcting these deleterious mtDNA mutations. Traditional CRISPR/Cas9 systems face challenges targeting mtDNA due to difficulties delivering guide RNA into mitochondria. However, innovative approaches are being developed to overcome these obstacles. One such method involves appending an RNA transport-derived stem-loop element to guide RNAs, facilitating their import into mitochondria and enabling precise editing of mtDNA. Additionally, mitochondrial base editing tools have been engineered to create precise nucleotide changes without causing double-stranded breaks. These tools have been utilized to establish novel disease models and hold potential for personalized gene therapies targeting mtDNA-associated disorders (Sahel et al. 2023; Poojitha Pinjala et al. 2024).

**Table 6:** Gene mutations that CRISPR systems have been used to edit in neurodegenerative diseases

Gene mutation	Associated disease	CRISPR editing approach	Reference
MT-ND1 and MT-ND2 mutations	Leigh syndrome	Development of mitochondrial base editing tools to correct mutations	Kar et al. (2023)
G13513A mutation in ND5 gene	MELAS and Leigh syndrome	TALEN-mediated shift of mitochondrial DNA heteroplasmy	
A53T mutation in SNCA gene	PD	CRISPR/Cas9-mediated correction of the A53T mutation	Nojadeh et al. (2023)
CAG repeat expansion in ATXN1 gene	Spinocerebellar ataxia type 1	Use of CRISPR/Cas9 nickase to induce contractions in expanded CAG repeats	
G8363A mutation in tRNA^Lys gene	Myoclonus epilepsy and ragged-red fiber (MERRF) syndrome	Development of mitochondrial base editing tools to correct mutations	Kar et al. (2023)

However, despite its favorable prospects for gene editing, the clinical application of CRISPR/Cas9 remains challenging due to issues such as immune response activation, off-target effects, and inefficient in vivo delivery (Alshial et al. 2023).

### Mitochondrial gene editing

Mitochondrial gene editing is a transformative approach to resolve mitochondrial dysfunction by precisely targeting and modifying mtDNA. Given the critical role of mitochondria in energy production and cellular metabolism, mutations in mtDNA can lead to severe genetic disorders (Kar et al. 2023). Recent advancements in gene editing technologies, particularly programmable deaminases and CRISPR-based systems, have enhanced the ability to edit mtDNA with high precision. For instance, engineered transcription activator-like effector-linked deaminases (TALEDs) have been developed to achieve targeted A-to-G and C-to-T edits in mtDNA, significantly reducing off-target effects and improving editing accuracy (Shoop et al. 2023). These innovations have enabled the creation of animal models for mitochondrial diseases such as Leigh syndrome, facilitating the study of disease mechanisms and potential therapies. Additionally, nuclease-based approaches leverage the mitochondria’s natural repair mechanisms, allowing for the elimination of mutant mtDNA by inducing double-strand breaks, which promotes the replication of wild-type mtDNA. The ability to manipulate mtDNA holds promise for treating mitochondrial diseases and provides insights into fundamental mitochondrial biology. As research progresses, mitochondrial gene editing may lead to effective therapies for various conditions associated with mitochondrial dysfunction (Yin et al. 2022).

## Mitotherapies

### Mitotherapy in AD

Electron microscopy has identified distinct alterations in the mitochondrial ultrastructure of AD patients compared to healthy controls (Yang et al. 2023). Recent findings suggest that Aβ accumulation plays a central role in triggering mitochondrial dysfunction in AD. Consequently, therapeutic strategies to enhance mitochondrial function and preserve mitochondrial integrity are gaining traction for treating neurological disorders, including AD (Cenini and Voos 2019). Emerging evidence supports the potential of exogenous mitochondria to infiltrate animal tissue cells for therapeutic purposes through local or intravenous delivery. Mitochondrial transfer, a natural process in the brain, involves neurons offloading damaged mitochondria to astrocytes for degradation and recycling. Astrocytes, in turn, can supply functional mitochondria for neuronal uptake. This physiological mechanism provides a robust foundation for mitotherapy for neurological conditions associated with mitochondrial dysfunction (Nascimento-dos-Santos et al. 2021).

The first study demonstrating the therapeutic potential of unmodified mitochondria in AD used a mouse model induced by intracerebroventricular injection of Aβ peptide. Administering freshly isolated human mitochondria from HeLa cells intravenously significantly improved cognitive function in AD mice, nearly bringing it back to levels seen in non-AD mice. The treated mice also showed less neuronal damage and gliosis in the hippocampus than untreated AD mice. Enhancements in mitochondrial function were observed, including increased activity of essential enzymes in energy metabolism, such as CS from the Krebs cycle and cytochrome c oxidase (COX), the fourth electron transport chain complex (Javadpour et al. 2024; Weidling and Swerdlow 2020).

### Mitotherapy in PD

Improving mitochondrial function through mitotherapy is a promising strategy for PD. Multiple studies have shown that infusing or transplanting freshly isolated mitochondria notably enhances mitochondrial functionality (Bhatt et al. 2024; Thomas and Beal 2010). These investigations revealed that mitochondrial transplantation not only elevated the expression of mitochondrial proteins but also increased the levels of precursor metabolites, thereby improving energy production and cellular respiration. Research involving cells that received transplanted mitochondria indicated reduced ROS levels and increased ATP production. Additionally, studies using the MPTP rat model after mitochondrial injection have demonstrated increased activation of the ETC, restoration of cellular apoptosis and necrosis processes, and reduced ROS levels. These results highlight the therapeutic potential of injecting mitochondria into the brain or administering them intravenously (Henrich et al. 2023). The concept of mitochondrial donation has been observed across various human and animal tissues. This process involves replacing dysfunctional mitochondria with healthy ones, representing a promising strategy for treating mitochondrial disorders. Mitochondrial donation has been linked to restoring damaged cells, improved OXPHOS activity, elevated ATP synthesis, and enhanced mitochondrial function. An innovative approach has recently gained attention, emphasizing the transplantation of cellular components, including mitochondrial organelles, to support cellular recovery and function. In vitro studies on the transplantation of xenogeneic mitochondria have indicated that this method can restore mitochondrial respiration in human cells lacking mitochondrial DNA (Zambrano et al. 2022).

The intranasal delivery of drugs offers a new strategy for targeting the brain and central nervous system, including conditions like PD. Traditional intravenous methods often face challenges in penetrating the BBB, whereas the intranasal route enhances drug development by facilitating access to the brain. Researchers propose that mitochondria may serve as an effective non-invasive delivery system for clinical applications, as this method allows for direct nose-to-brain transport without requiring mitochondrial modification. Extensive research has demonstrated that transferring mitochondria from stem cells to damaged cells can boost ATP production, restore mitochondrial function, and protect recipient cells from apoptosis. The success of mitochondrial transplantation is primarily influenced by the source and quality of the transplanted mitochondria, which are crucial for efficient neural uptake in the brain, as shown by well-established cellular mechanisms that aid in incorporating additional mitochondria. Research on the intranasal delivery of mitochondria revealed that mitochondrial complex I protein respiration in SN neurons was significantly lower (52%) compared to local injection (85%) when identical treatment frequencies and intervals were used; however, the improvement in the survival of dopaminergic neurons in the nigra was similar (Wen and Ren 2024). MSCs, derived from various tissues, have gained significant attention for their regenerative and immunomodulatory properties in treating different diseases. Their ability to transfer mitochondria plays a key role in cellular function, affecting biological processes in normal and pathological conditions. As a cornerstone of stem cell therapies, MSCs are emerging as an innovative strategy for tissue regeneration (Velarde et al. 2022). Growing evidence underscores their ability to directly donate mitochondria, aiding cellular recovery after injury and reducing tissue degeneration linked to mitochondrial dysfunction. Typically, MSCs are isolated from sources like bone marrow or adipose tissue, and they can also be derived from induced pluripotent stem cells (Velarde et al. 2022).

## Optogenetic induction of the mitochondria

**Fig. 13:**
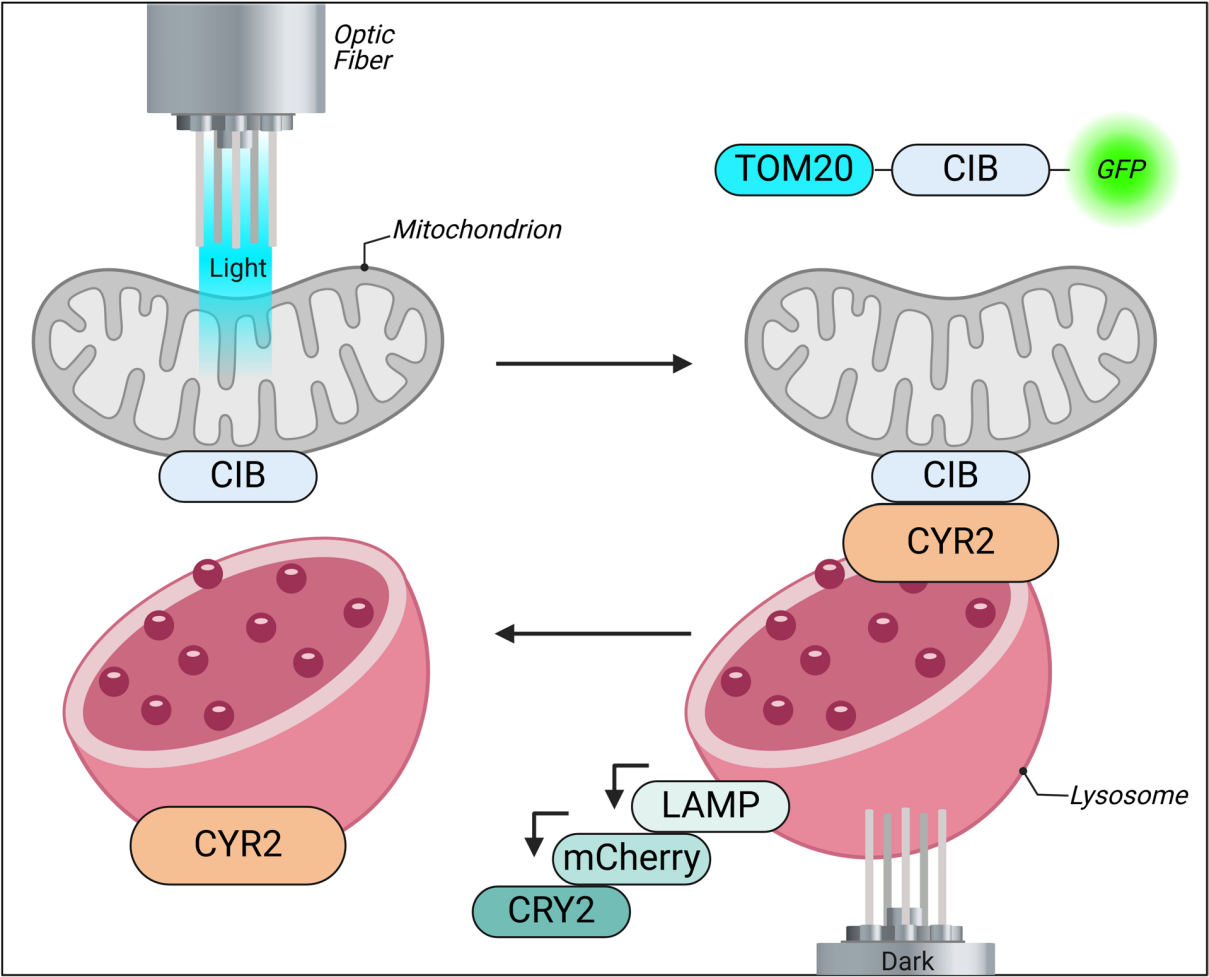
Optogenetics triggers mitochondria-lysosome contacts (MLCs) by utilizing light-sensitive proteins such as CRY2 and CIB, which are anchored to lysosomes and mitochondria through LAMP and TOM20 domains, with GFP and mCherry serving as expression markers. All figures have been created using BioRender.com

Optogenetic induction of mitochondria represents a groundbreaking approach to manipulating mitochondrial dynamics with high precision. This technique utilizes light-sensitive proteins to control mitochondrial processes such as fission and mitophagy, which are crucial for maintaining cellular health. For instance, a novel optogenetic tool has been developed that enables the reversible induction of mitophagy by recruiting the pro-autophagy protein AMBRA1 to the mitochondrial surface upon blue light exposure. This method has demonstrated effectiveness in various models, including HeLa cells and zebrafish embryos, showcasing its potential for therapeutic applications in neurotoxicity and other conditions where mitochondrial dysfunction is prevalent (Strappazzon et al. 2019) (Fig. 13).

Moreover, real-time optogenetic control of mitochondrial fission has been achieved by establishing mitochondria-lysosome contacts through light-induced dimerization. This method allows researchers to study mitochondrial fission rates and restore mitochondrial functions in cells with genetic defects, thereby improving cellular metabolism and ATP production (Zhang et al. 2025). The ability to induce mitochondrial aggregation and fusion through optogenetic systems further highlights the versatility of this approach, offering new avenues for addressing diseases linked to oxidative stress and mitochondrial fragmentation.

### Limitations of optogenetic induction of mitophagy

Optogenetics technology has shown potential in mitochondrial-based therapies. However, translating these approaches from in vitro or small animal models to humans presents several challenges. A high-intensity light is often required to activate optogenetic proteins like channelrhodopsin-2 (ChR2), which may induce oxidative stress and harm cells (Ernst et al. 2019). Additionally, proper localization of these proteins is crucial for effectiveness, as mislocalization can reduce efficacy or cause toxicity (D’ Amato et al. 2023; Hinton et al. 2024; Lu and Jiang 2024; Ma et al. 2020). While rodent models are commonly used, their differences from human retinal structures limit direct translation, and larger animal models, such as non-human primates, pose ethical and financial challenges (Pinjala et al. 2023; Qiu et al. 2022). Furthermore, although optogenetics offers precise control over mitochondrial dynamics like fission, the low penetrability of blue light into deep tissues restricts its broader application (McClements et al. 2020; Borchert et al. 2024). Researchers are developing more sensitive optogenetic tools to address these limitations, improving delivery methods through advanced viral vectors, and studying larger animal models that better replicate human conditions (Borchert et al. 2024).

## Other effects of light on the mitochondria

**Fig. 14:**
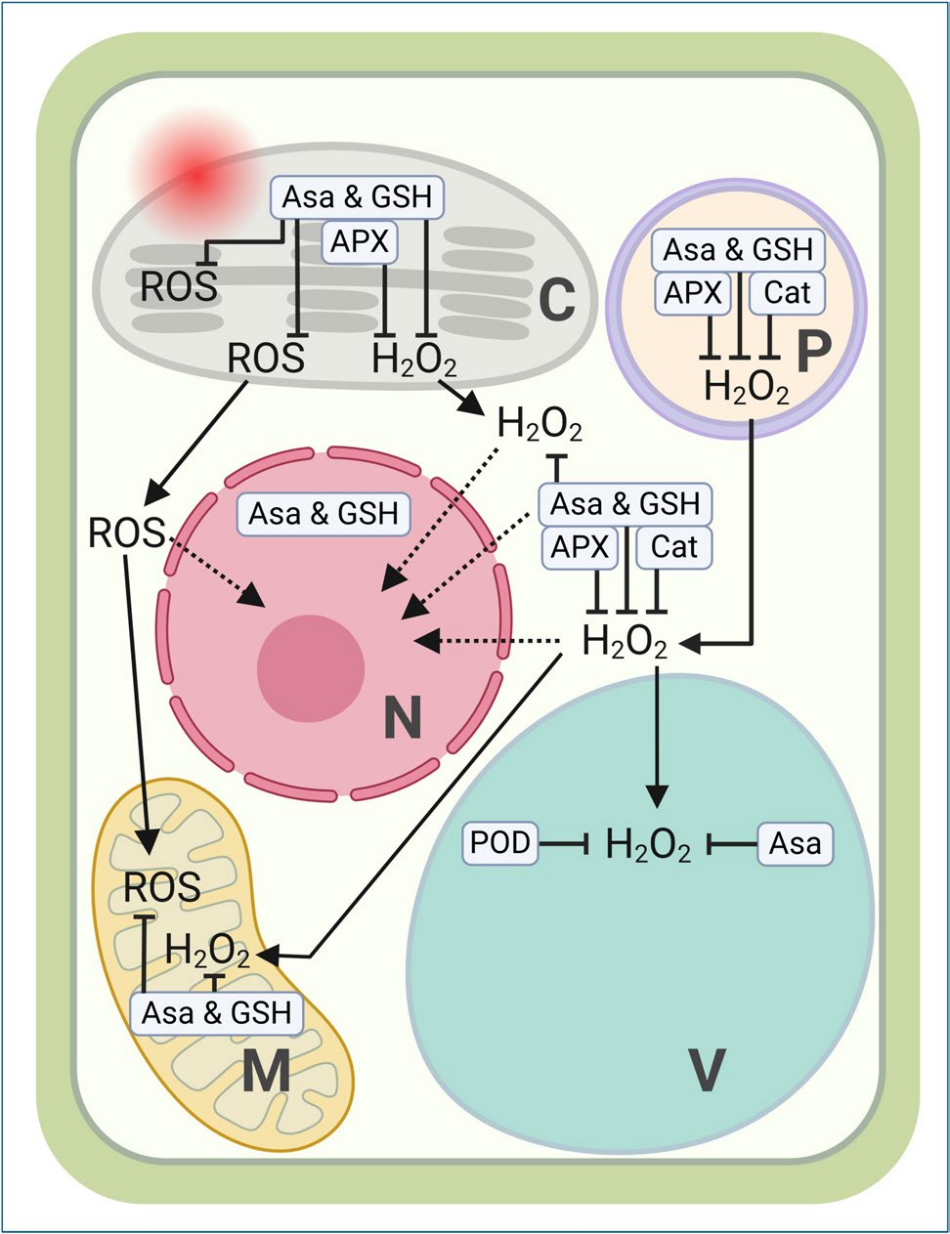
The figure examines the impact of excessive exposure to white and far-red light on the subcellular generation of ROS, focusing on hydrogen peroxide (H_2_O_2_) and its detoxification mechanisms within specific cellular compartments. These detoxification processes involve antioxidants such as ascorbate (AsA), glutathione (GSH), and enzymatic systems. A conceptual diagram illustrates the proposed model, highlighting the effects of excess white light (represented by a white thunderbolt) and far-red light (depicted as a dark red thunderbolt) on the subcellular accumulation of H_2_O_2_ and other ROS. The model also indicates potential signaling pathways using dotted arrows. Key components include ascorbate peroxidase (APX), catalase (Cat), chloroplast (C), mitochondria (M), nucleus (N), peroxisome (P), guaiacol-type peroxidase (POD), and vacuole (V)

Excessive exposure to white and far-red light can significantly influence the production of ROS in plant cells, particularly hydrogen peroxide (H_2_O_2_) (Borbély et al. 2022). This phenomenon is crucial as H_2_O_2_ accumulation can lead to oxidative stress, damaging cellular components (Hossain et al. 2015; Johri and Beal 2012a, b). Plants utilize compartment-specific detoxification mechanisms involving antioxidants like ascorbate (AsA) and glutathione (GSH), along with enzymatic systems such as ascorbate peroxidase (APX), to mitigate ROS effects (Fig. 14) (Du and Yan 2010; Zhou et al. 2022). Understanding these processes is vital, especially in the context of neurodegenerative diseases, where mitochondrial dysfunction and oxidative stress are implicated. Mitochondrial-based therapies targeting ROS (Borbély et al. 2022) may offer innovative neuroprotection and cellular health strategies in these conditions (Van Brenk et al. 2024).

Additionally, light exposure, mainly through photobiomodulation (PBM), has significantly influenced mitochondrial dysfunction in neurons, a critical factor in neurodegenerative diseases. PBM, utilizing specific wavelengths such as 670 nm and 810 nm, enhances mitochondrial energy metabolism, leading to increased oxidative phosphorylation and ATP production, which is vital for neuronal health (Gomes et al. 2023; Trajano et al. 2024; Fear et al. 2023). This therapeutic approach has demonstrated efficacy in restoring synaptic plasticity and mitigating cognitive decline in AD models by reducing ROS accumulation and improving mitochondrial function (Fear et al. 2023; Ravera et al. 2025). Additionally, light therapy can modulate mitochondrial dynamics, potentially reversing dysfunction associated with various neurodegenerative conditions (Fear et al. 2023). However, excessive light exposure can also induce mitochondrial stress and ROS production, highlighting the need for careful application (Fear et al. 2023).

## The role of mitochondrial-derived vesicles (MDVs) in neurodegenerative disease

Mitochondrial-derived vesicles (MDVs) are emerging as a promising therapeutic strategy in treating neurodegenerative diseases, mainly due to their role in addressing mitochondrial dysfunction, a hallmark of conditions like AD and PD. Recent studies highlight the potential of engineered exosomes, such as those derived from activated neutrophils, to enhance mitochondrial function and mitigate neurotoxicity associated with amyloid-beta in Alzheimer’s disease (Zhang et al. 2024). Additionally, therapies leveraging mesenchymal stem cells and their secretions can facilitate mitochondrial transfer to restore cellular health in affected neurons (Ore et al. 2024). The focus on mitochondrial dynamics, including fission and fusion processes, alongside the use of mitochondria-targeted antioxidants, presents novel avenues for drug development aimed at reducing oxidative stress and improving mitochondrial integrity (Choi et al. 2024; Yan et al. 2023b, a).

### Challenges for mitochondrial-based treatments for neurodegenerative illnesses

Mitochondrial-based medicines for neurodegenerative diseases confront a few novel challenges (Table 7). Firstly, the complexity of mitochondrial dysfunctionality, which incorporates bioenergetics, disabled biogenesis, and disturbed flow, complicates improving new targeted therapies (Yusoff and Khair, 2024). Moreover, whereas inventive approaches such as mitochondrial transplantation and quality altering (e.g., mito-CRISPR) appear guaranteed, they raise concerns concerning security, counting potential tumorigenicity, and off-target effects (Ore et al. 2024). Moreover, the regenerative capacity of neurons is restricted, making it difficult to reestablish work once serious harm has occurred (Kalani et al. 2023).

**Table 7:** Adverse effects of mitochondrial therapies

Mitochondrial therapy	Potential toxicities/adverse effects
Mitochondrial-targeted antioxidants	Possible pro-oxidant effects at high concentrations, leading to cellular damage.
PGC-1α activators	Risk of excessive mitochondrial biogenesis, which may lead to tumorigenesis and metabolic imbalance.
Exercise mimetics	Side effects related to metabolic dysregulation and the potential for off-target effects.
Mitophagy modulation	Disturbance in mitochondrial quality control, potentially leading to cellular stress and apoptosis.
PINK/Parkin drugs	Possible interference with normal mitochondrial functions leads to neurodegenerative effects.
Mitochondrial transplantation	Rejection of transplanted mitochondria, immune response, and potential long-term effects on cellular function.
CRISPR-Cas9 gene editing	Off-target effects, unintended mutations, and potential harm to non-target DNA.
Mitochondrial gene editing	Risk of causing mutations or mitochondrial dysfunction if not adequately controlled.
Mitotherapy	Mitochondrial overload, imbalance in mitochondrial dynamics, and potential organ toxicity.
Optogenetics induction	Risk of inducing cellular stress, potential for seizures, and inflammation.

The dependence on antioxidant medications to moderate ROS presents challenges, as these treatments have had constrained victory in clinical trials (Table 8) (Kalani et al. 2023). Further rigorous preclinical studies, including those using human cell models and larger animal models, are necessary to validate the efficacy and safety of mitochondrial-based therapies before human trials.

**Table 8:** Current therapeutic drugs targeting neurodegenerative diseases and their clinical stages

Therapy	Disease	Mechanism	Clinical stage
Coenzyme Q10 (Ubiquinone)	PD	Enhances mitochondrial ETC, antioxidant effect	Clinical trials (Phase II)
Creatine	ALS	Improves mitochondrial function, prevents cell death	Approved (ALS)
MitoQ	AD	Antioxidant targeting mitochondria, reduces oxidative stress	Clinical trials (Phase II)
L-carnitine	PD, AD	Enhances fatty acid oxidation and ATP production	Approved (Alzheimer’s)
Alpha-lipoic acid (ALA)		Antioxidant, improves mitochondrial function, reduces oxidative stress	Approved
EPI-743 (Vatiquinone)	Leigh syndrome, PD	Enhances mitochondrial function by increasing energy production	Clinical trials (Phase III)
Elamipretide (Stealth BioTherapeutics)	AD, ALS	Restores mitochondrial function, reduces oxidative stress and apoptosis	Clinical trials (Phase III)
Methylene blue	AD	Inhibits mitochondrial complex IV, improves mitochondrial respiration	Clinical trials (Phase II)
Idebenone	Friedreich’s ataxia	Coenzyme Q10 analogue, enhances mitochondrial ATP production	Approved (Friedreich's ataxia)
Raxone (Idebenone)	Leber’s hereditary optic neuropathy	Reduces mitochondrial oxidative damage and improves ATP production	Approved
N-acetylcysteine (NAC)	PD	Antioxidant, improves mitochondrial glutathione levels	Clinical trials (Phase II)
Metformin		Improves mitochondrial function, reduces oxidative stress, and improves glucose metabolism	
Rapamycin	AD, PD	Inhibits mTOR signaling, improves mitochondrial autophagy	
Pterostilbene	AD	Activates SIRT1 pathway, enhances mitochondrial biogenesis	Preclinical
Resveratrol	AD, PD	Activates SIRT1, improves mitochondrial biogenesis and oxidative stress reduction	Clinical trials (Phase II)
Salsalate	AD	Reduces inflammation, improves mitochondrial function	
Bortezomib	ALS	Reduces protein aggregation, restores mitochondrial function	
AICAR	HD	Activates AMP-activated protein kinase (AMPK), improves mitochondrial function	Preclinical
Caffeine	PD	Improves mitochondrial respiration, neuroprotective effect	Approved
Resveratrol and pterostilbene combination	AD	Dual activation of SIRT1 pathway for enhanced mitochondrial function	Preclinical
N-Acetyl-L-cysteine (NAC)	AD, PD	Restores mitochondrial glutathione, reduces oxidative stress	Clinical trials (Phase II)
Cyclophilin D inhibitors	AD, ALS	Modulates mitochondrial permeability transition pore, protects mitochondrial integrity	Preclinical
Sildenafil (Viagra)	PD	Enhances mitochondrial function by increasing nitric oxide signaling	Clinical trials (Phase II)
Urolithin A	AD, PD	Enhances mitophagy (clearance of dysfunctional mitochondria)	
Mitochondrial transplantation	Various neurodegenerative diseases	Transplantation of healthy mitochondria to damaged tissues	Preclinical
BHB (Beta-Hydroxybutyrate)	PD, AD	Provides alternative energy source, supports mitochondrial function	Clinical trials (Phase II)
NAD+ precursors		Restores NAD+ levels, improving mitochondrial function and energy production	
Pramipexole	PD	Mitochondrial protection, enhances dopaminergic function	Approved (Parkinson’s)
Pioglitazone	PD, AD	Improves mitochondrial dynamics, reduces oxidative stress	Clinical trials (Phase II)
Ginkgo biloba	AD	Increases ATP production, improves mitochondrial energy metabolism	Approved
Nilotinib	PD	Reduces alpha-synuclein aggregation, protects mitochondria	Clinical trials (Phase II)
Curcumin	PD, AD	Reduces mitochondrial oxidative stress, increases mitochondrial biogenesis	Clinical trials (Phase II)
Trimetazidine		Enhances mitochondrial oxidative metabolism, improves ATP production	Approved
Ferulic acid	PD	Antioxidant properties, improves mitochondrial bioenergetics	Preclinical
Bimoclomol	ALS	Enhances mitochondrial heat shock protein (HSP) expression, protects neurons	Clinical trials (Phase II)
Lithium	PD, AD	Enhances mitochondrial biogenesis and reduces oxidative stress	Clinical trials (Phase II)
Pifithrin-α	PD	Modulates mitochondrial stress response pathways	Preclinical
Butylated hydroxytoluene (BHT)		Antioxidant, protects mitochondrial membranes from oxidative damage	
Sodium oligomannate		Reduces neuroinflammation, improves mitochondrial function	Clinical trials (Phase III)
Deferiprone	PD, AD	Reduces iron accumulation in mitochondria, reduces oxidative stress	

## Future perspectives and conclusions

Advancements in cell therapy, including stem cell-based methods, gene-modified therapies, and exosome-based approaches, offer great promise in revolutionizing the treatment of neurodegenerative diseases. Mitochondria, crucial organelles responsible for energy production, immune responses, neurotransmitter synthesis, and calcium buffering, are linked to several severe conditions, including neurodegenerative diseases like Alzheimer’s and Parkinson’s and cardiovascular and metabolic disorders like diabetes. Understanding mitochondrial biology and medicine is vital for developing effective therapeutic strategies targeting these diseases’ root causes. Improving mitochondrial health should be approached in a context-specific manner, considering both disease and tissue specificity while accounting for mitochondrial heterogeneity within tissues to understand its implications for health and disease fully.

Mitochondrial heterogeneity, arising from distinct subpopulations with specialized functions, is linked to age-related decline, neurodegenerative diseases, metabolic disorders, and cancer. Exploring how mitochondrial diversity affects cellular function could uncover new therapeutic targets and approaches to preserve mitochondrial health and tissue balance. For example, targeting alpha-synuclein in Parkinson’s disease and amyloid-β in Alzheimer’s disease may help alleviate mitochondrial dysfunction. Continued research and interdisciplinary collaboration are crucial for progressing in mitochondrial biology and addressing neurodegenerative diseases.

In conclusion, the advancements in CRISPR/Cas9 and mitochondrial gene editing technologies hold significant promise for addressing the challenges posed by neurodegenerative disease. The ability to precisely modify genetic material and target mitochondrial dysfunction may pave the way for innovative therapeutic approaches that restore cellular function and improve patient outcomes. As research progresses, the potential of these strategies to not only correct genetic abnormalities but also enhance mitochondrial health presents an exciting frontier in medicine. However, challenges such as off-target effects and delivery mechanisms must be carefully navigated to realize the full clinical potential of these groundbreaking technologies. Ongoing investigations and clinical trials will be crucial in validating the efficacy and safety of these interventions, bringing hope to those affected by these debilitating conditions.

## Data Availability

All source data for this work (or generated in this study) are available upon reasonable request.
